# Discrepancy in interactions and conformational dynamics of pregnane X receptor (PXR) bound to an agonist and a novel competitive antagonist

**DOI:** 10.1016/j.csbj.2022.06.020

**Published:** 2022-06-13

**Authors:** Azam Rashidian, Enni-Kaisa Mustonen, Thales Kronenberger, Matthias Schwab, Oliver Burk, Stefan A. Laufer, Tatu Pantsar

**Affiliations:** a Department of Internal Medicine VIII, University Hospital Tuebingen, Otfried-Müller-Strasse 14, Tuebingen DE 72076, Germany; b Dr. Margarete Fischer-Bosch-Institute of Clinical Pharmacology, Stuttgart and University of Tuebingen, Tuebingen, Germany; c Department of Pharmaceutical and Medicinal Chemistry, Institute of Pharmaceutical Sciences, Eberhard-Karls-Universität, Tuebingen, Auf der Morgenstelle 8, Tuebingen 72076, Germany; d Departments of Clinical Pharmacology, and Pharmacy and Biochemistry, University of Tuebingen, Tuebingen, Germany; e Cluster of Excellence iFIT (EXC 2180) “Image-Guided and Functionally Instructed Tumor Therapies”, University of Tuebingen, Tuebingen 72076, Germany; f Tuebingen Center for Academic Drug Discovery & Development (TüCAD2), Tuebingen 72076, Germany; g School of Pharmacy, Faculty of Health Sciences, University of Eastern Finland, Yliopistonranta 1, Kuopio 70210, Finland

**Keywords:** Pregnane X receptor, Molecular dynamics simulation, PXR ligand binding domain, SR12813 (PubChem CID: 446313), BAY-1797 (PubChem CID: 124125214)

## Abstract

Pregnane X receptor (PXR) is a nuclear receptor with an essential role in regulating drug metabolism genes. While the mechanism of action for ligand-mediated PXR agonism is well-examined, its ligand-mediated inhibition or antagonism is poorly understood. Here we employ microsecond timescale all-atom molecular dynamics (MD) simulations to investigate how our newly identified dual kinase and PXR inhibitor, compound 100, acts as a competitive PXR antagonist and not as a full agonist. We study the PXR ligand binding domain conformational changes associated with compound 100 and compare the results to the full agonist SR12813, in presence and absence of the coactivator. Furthermore, we complement our research by experimentally disclosing the effect of eight key-residue mutations on PXR activation. Finally, simulations of P2X4 inhibitor (BAY-1797) in complex with PXR, which shares an identical structural moiety with compound 100, provide further insights to ligand-induced PXR behaviour. Our MD data suggests ligand-specific influence on conformations of different PXR-LBD regions, including α6 region, αAF-2, α1-α2′, β1′-α3 and β1-β1′ loop. Our results provide important insights on conformational behaviour of PXR and offers guidance how to alleviate PXR agonism or to promote PXR antagonism.

## Graphical abstract

**Figure:**
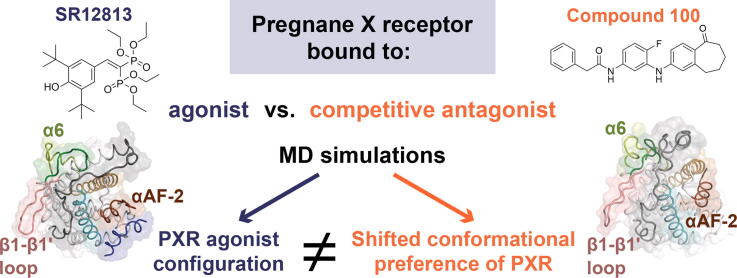

## 1 Introduction

Pregnane X receptor (PXR), also known as nuclear receptor (NR) subfamily 1 group I member 2, encoded by the gene *NR1I2*, is a ligand-dependent transcriptional factor that is activated by a structurally diverse set of small molecules [1]. PXR binds various xenobiotic compounds, such as endocrine-disrupting chemicals and pharmaceutical drugs, and endogenous ligands, such as hormones. Ligand-bound PXR regulates the transcription of genes encoding phase I and phase II drug metabolizing enzymes [2] as well as uptake and efflux transporters [[3], [4]]. PXR activation has an important role in drug-drug interactions (DDIs) [5], adverse drug reactions [6] and drug treatment efficacy [[4], [7], [8]]. In this regard, regulatory agencies, including the European Medicines Agency (EMA) [9] and the United States Food and Drug Administration (FDA) [10], have introduced *in vitro* assays for PXR activation and *in vivo* CYP expression levels in their pipelines for evaluation of drug safety. In addition to its role in small molecule metabolism, PXR is involved in regulation of diverse cellular processes including energy homeostasis, cell proliferation and inflammation [[11], [12]]. PXR expression adapts to (patho) physiological [13] and environmental stimuli [14].

**Fig. 1:**
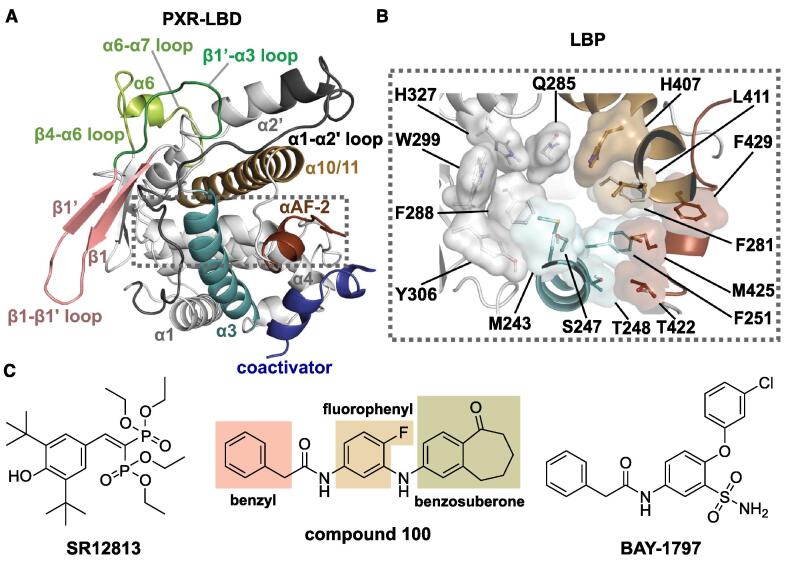
Overview of the PXR-ligand binding domain (LBD) structure and the small molecule ligands included in this study. (A) Crystal structure of PXR-LBD (PDB ID: 1NRL [22]). The regions of interest are highlighted with the following colour scheme that is used throughout this article: α1-α2′ loop (residues 177–198), dark grey; β-β1′ (residues 211–225), pink; β1′-α3 loop (residues 226–234), dark green; α3-helix (residues 240–260), cyan; β4-α6 loop (residues 309–314), α6 (residues 315–318) and α6-α7 loop (residues 319–323), light green; α10/α11 (residues 389–417) light brown; αAF-2 (residues 423–434), dark brown; coactivator peptide (SRC-1), blue. The dashed rectangular area denotes the location of the ligand binding pocket. (B) Ligand-binding pocket (LBP) of PXR. The main residues forming the LBP and participating in ligand binding are depicted in stick model with transparent molecular surface. Residues are coloured according to their respective regions (see A). (C) 2D structures of PXR agonist SR12813 (SRL), our in-house kinase inhibitor (compound 100), which was found to act also as a competitive PXR antagonist [42], and P2X4 antagonist BAY-1797 that is a PXR agonist [43]. Structure of compound 100 includes a benzosuberone moiety, a fluorophenyl ring and a benzyl group (structural moieties that are discussed in the results). (For interpretation of the references to colour in this figure legend, the reader is referred to the web version of this article.)

PXR structure comprises three domains: DNA binding domain (DBD), hinge region and ligand-binding domain (LBD). PXR interacts with the gene promoter region of target genes in DNA via its N-terminal DBD [15]. The hinge region is reported to be target of post-translational modifications, which affect PXR mediated gene regulation. For instance, acetylation and deacetylation of K109 modulates PXR transcription activity [16]. LBD comprises eleven α-helices, in addition to the αAF-2 helix (also known as activation function-2), and five-stranded β-sheet (Fig. 1A). The eleven α-helices form three aligned groups: α1/α3, α4/α5/α8/α9 and α7/α10/α11. PXR lacks the typical stable α2 and α6 helices that are found in other NRs [17]. With other NRs the ordered α6 helix results in more tightly packed smaller LBD [[17], [18]]. Moreover, while many NRs exhibit a three-stranded β-sheet in their LBD, PXR comes with two additional strands. These extra β-sheets, together with the additional α2′, contribute to a larger and more flexible LBD, when compared to other NR-LBDs [[19], [20], [21]]. This increased flexibility allows accommodation of diverse set of ligands to the ligand binding pocket (LBP) (Fig. 1B), which is a unique characteristic of PXR.

PXR heterodimerizes with retinoid X receptor alpha (RXRα) in cell cytoplasm and this complex (PXR-RXRα) is transported to the nucleus [23]. Binding an activator ligand to PXR-RXRα heterodimer in nucleus allows the exchange of cofactor (release of a corepressor and recruitment of a coactivator) [24]. Subsequently, this activated PXR complex regulates expression of the target gene.

While the knowledge of PXR ligand-induced activation is well established, the mechanism of action for ligand-mediated inhibitory/antagonistic effects on PXR is less understood [25]. Despite of the numerous PXR agonists that have been reported, a very limited number of antagonists exist. Antagonists binding to the LBP include sulforaphane [26], and SPA70 [27] as shown by *in vitro* ligand-binding competition assays. Other PXR antagonists, such as ketoconazole or FLB-12 [28] can reduce the endogenous PXR activation without directly or exclusively binding to the LBP, whereas coumestrol [[29], [30]] shown to bind to the LBP and AF-2 domain. What renders a PXR ligand an agonist or an antagonist, and their respective structural triggers, remains poorly understood. For instance, Lin et al. discovered that a close analogue of SPA70, SJB7, is a PXR agonist [27]. Structural differences between SPA70 and SJB7 are minimal, it only exists in their terminal aromatic ring substituents (SI Fig. S1). Lin et al. hypothesized that SJB7 interacts through its *p*-methoxy group with a hydrophobic spot on αAF-2 (residues L428 and F429) and stabilizes the αAF-2 to enable interaction with a coactivator, while SPA70 fails in this due to the lack of this group. More recently, Li et al. reported a set of SPA70 analogues, revealing diverse biological activities of these ligands, ranging from agonists to antagonists and partial agonists [31]. This exemplifies how subtle structural changes may completely shift PXR ligand function and it highlights the promiscuity of PXR-LBD.

No co-crystal structures of PXR and antagonist are currently available to elucidate the details of the PXR–antagonist interactions. Also, for instance, docking approaches are limited as they are unable to capture PXR’s characteristic flexibility [[21], [32], [33], [34], [35], [36]] and the effect of water [37]. Therefore, molecular dynamics (MD) simulations have been utilized for better understanding of transition state of active to inactive in nuclear receptors [38]. Previous PXR-related MD simulations have mainly focused on PXR agonists. Chandran et al. studied the dynamic behavior of PXR-LBD apo structure in comparison to the agonist-bound state, using short MD simulations of 100 ns [39]. They could identify several conformational states for apo PXR-LBD with different volume through MD simulation. Their study revealed that the SR12813 agonist binding events restrict the LBD in a conformation relevant to the size and shape of the ligand. Further, Motta et al. employed the MD-binding method to simulate the SR12813 entry into the LBP. Their result suggested that the ligand would enter to the LBP via a channel between α2 and α6 helices [40]. They also implemented scaled MD (SMD) simulations to extend the sampling of the bound conformations of SR12813 within PXR-LBD (with a total of 2 µs). They predicted that the binding mode of SR12813 observed in the crystal structure PDB ID: 1NRL [22] is the most stable one among other available PXR-SR12813 complex structures. In addition, Huber et al. performed 200 ns MD simulations of wild type (WT) PXR-LBD and W299A mutant, without ligand and with T090131713 (agonist), SPA70, and SJB7 [41]. They suggested that the extra space conferred by the W299A is the reason for the observed antagonist-to-agonist switch with this mutant for SPA70. This extra space let SPA70 to reside deeper in the pocket, preventing the αAF-2 dislocation and maintaining PXR active.

We recently discovered a novel competitive PXR antagonist from the Tübingen kinase inhibitor collection (TüKIC) compound library [42]. This competitive antagonist, compound 100 (Fig. 1C), suppresses both rifampicin- and SR12813-induced PXR activation, and it does not induce recruitment of SRC-1 to PXR in coactivator recruitment mammalian 2-hybrid assay [42]. Here we aimed to disclose why this kinase inhibitor acts also as an antagonist when in complex with PXR and how it differs from a typical agonist (SR18213). To this end, we applied microsecond timescale MD simulations to understand how these ligands influence PXR’s conformational dynamics. Furthermore, we experimentally tested a selection of PXR-LBD mutants and their influence on PXR activity. Finally, we simulated PXR-LBD in complex with BAY-1797 [43], a weak PXR agonist, that shares structural similarity to our competitive antagonist. Our results highlight ligand-specific dynamical behaviour of PXR-LBD and suggest key-changes in ligand-induced antagonism.

## 2 Results

### 2.1 Microsecond timescale molecular dynamics simulations reveal discrepancy in motions of compound 100 and SR12813 bound PXR-LBD

To investigate and to compare the interactions and conformational dynamics of the novel competitive antagonist and the classical agonist, we conducted a total of 60 µs unbiased all-atom MD simulations. These simulations comprised three systems: PXR-LBD in complex with compound 100 (C-100; total simulation time of 30 µs), SR12813 (SRL; 20 µs) and SR12813 together with SRC-1 coactivator peptide (SRL + Co; 10 µs) (SI Fig. S13).

**Fig. 2:**
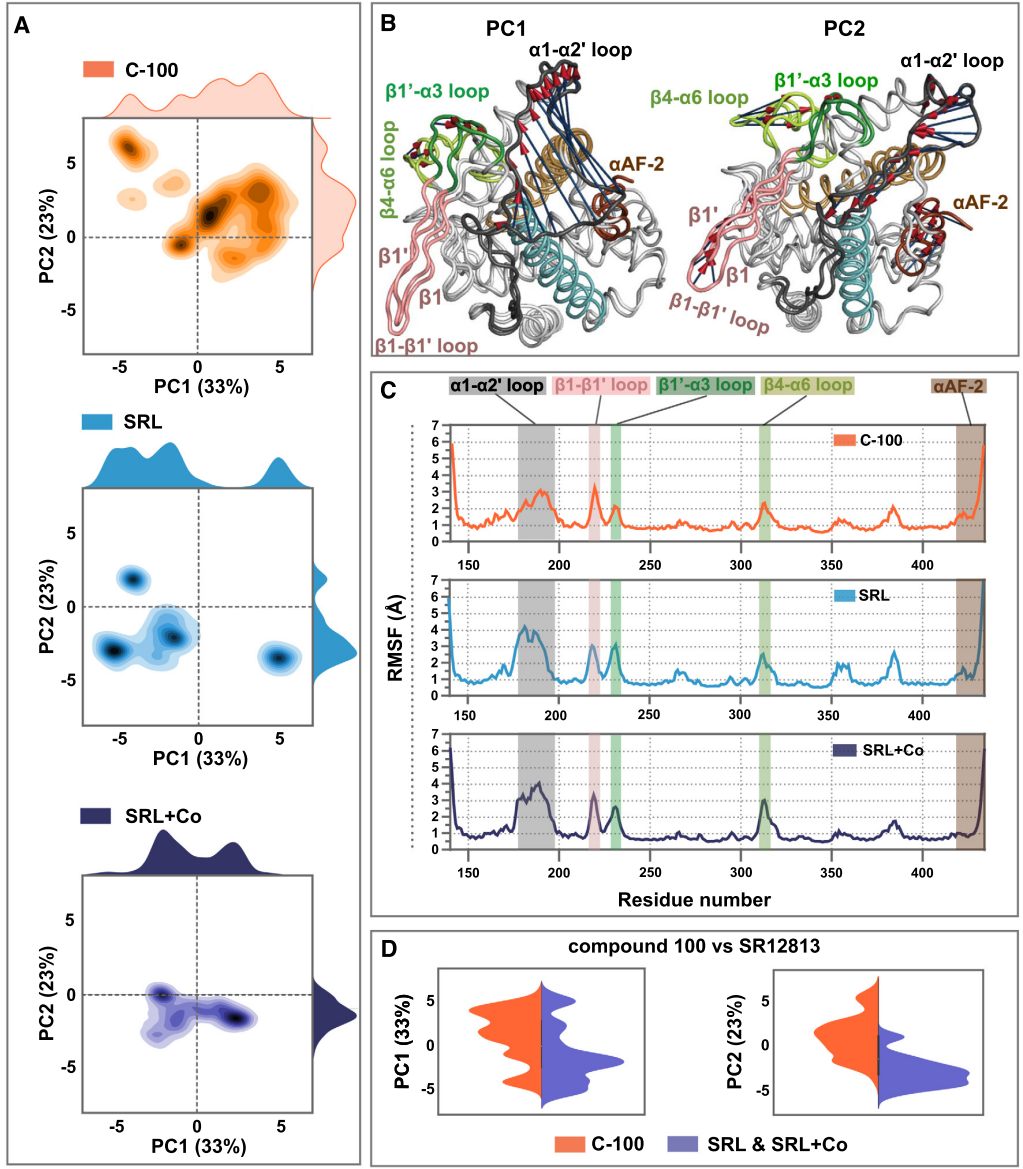
Conformational dynamics of PXR-LBD is different in compound 100 and SR12813 bound systems. (A) Principal component analysis (PCA) score plot of PC1 and PC2. Each system of the joint PCA is depicted separately: PXR in complex with compound 100 (C-100), orange; PXR in complex with SR12813 (herein called SRL), light blue; PXR in complex with SR12813 and SRC-1 coactivator peptide (herein called SRL + Co), dark blue. (B) The extreme movements of PC1 (left) and PC2 (right). Protein is colour coded as in Fig. 1A. Extreme movements related to each principal component are highlighted with purple arrows with red cones that indicate the direction of the movement. (C) Root-mean-square fluctuation (RMSF) of the protein backbone. The regions associated with the PCA extreme movements are highlighted with their respective colours as in B. (D) A joint comparison of the distributions of PC1 and PC2 scores between C-100 and combined agonist systems (SRL and SRL + Co) shown in violin plot. A kernel density estimation is applied to display the distribution of the data. C-100 is shown in light orange and combined agonist systems shown in purple colour. (For interpretation of the references to colour in this figure legend, the reader is referred to the web version of this article.)

First, to gain a better understanding of the PXR-LBD dynamics, we conducted principal component analysis (PCA) to identify the most essential motions of the protein. The first and the second principal components (PCs) describe together 56% of the data (PC1: 33% and PC2: 23%), while other PCs exhibit individual contributions below 10% (SI Table S1); thus, we focused our analysis on PC1 and PC2 (Fig. 2). The most extensive motions of PC1 occur in α1-α2′ loop (Fig. 2B; SI Movie M1). In addition, minor movement is observed in the β4-α6 loop (N-terminus of α6 region) and β1′-α3 loop. Of note, these regions, excluding the β4-α6 loop, are part of the novel insert of PXR-LBD that is not found in other NRs. This feature enables PXR-LBD to bind to a wide range of ligands [19]. PC2 displays extensive movement in β4-α6 loop, αAF-2 region, and the α1-α2′ loop (Fig. 2B; SI Movie M2). In addition, minor movement is observed in β1-β1′ loop and β1′-α3 loop. Importantly, these regions identified by PCA agree with the overall dynamical behaviour of the protein, as demonstrated by backbone root-mean-square fluctuations (RMSFs) (Fig. 2C). Overall, the trend in these fluctuations agrees with the B-factors of the PXR crystal structures (SI Fig. S3). The highest fluctuations appear in α1-α2′ loop, β1-β1′loop, β1′-α3, β4-α6 loop and αAF-2 region. Here, system specific differences are evident. In PCA highlighted regions, C-100 exhibits lower RMSF values in α1-α2′ loop, β4-α6 loop and β1′-α3 loop, and higher values in αAF-2 region, compared to the other systems (Fig. 2C; SI Table S2). The least fluctuation in the αAF-2 region is observed in SRL + Co.

Next, we shifted our focus on the differences in the PC scores among the systems. PC1 scores of the compound 100 and SR12813 bound systems are distributed in a wide range (Fig. 2A). However, higher values of PC1 are observed with C-100 (Fig. 2A, SI Fig. S2). Even greater difference is seen with PC2, where C-100 displays clearly higher values. Furthermore, a joint comparison of the PC scores between C-100 and combined agonist systems exemplifies the observed differences (Fig. 2D). Generally, higher PC1 scores are observed for the C-100 compared to the agonist systems (medians of 1.56 Å and −1.77 Å for C-100 and agonists, respectively). Differences in PC2 are more evident (medians of 1.75 Å and −2.77 Å for C-100 and agonists, respectively). Interestingly, PC2 represents the dislocation of αAF-2 from α3-helix (Fig. 2B; SI Movie M2), a movement which is associated with PXR antagonism [41], and β4-α6 loop association to α2′ (Fig. 2B; SI Movie M2). Overall, PCA exemplifies a clear ligand-dependent conformational behaviour of PXR-LBD.

### 2.2 Conformational behaviour of the α6 region is ligand-dependent

**Fig. 3:**
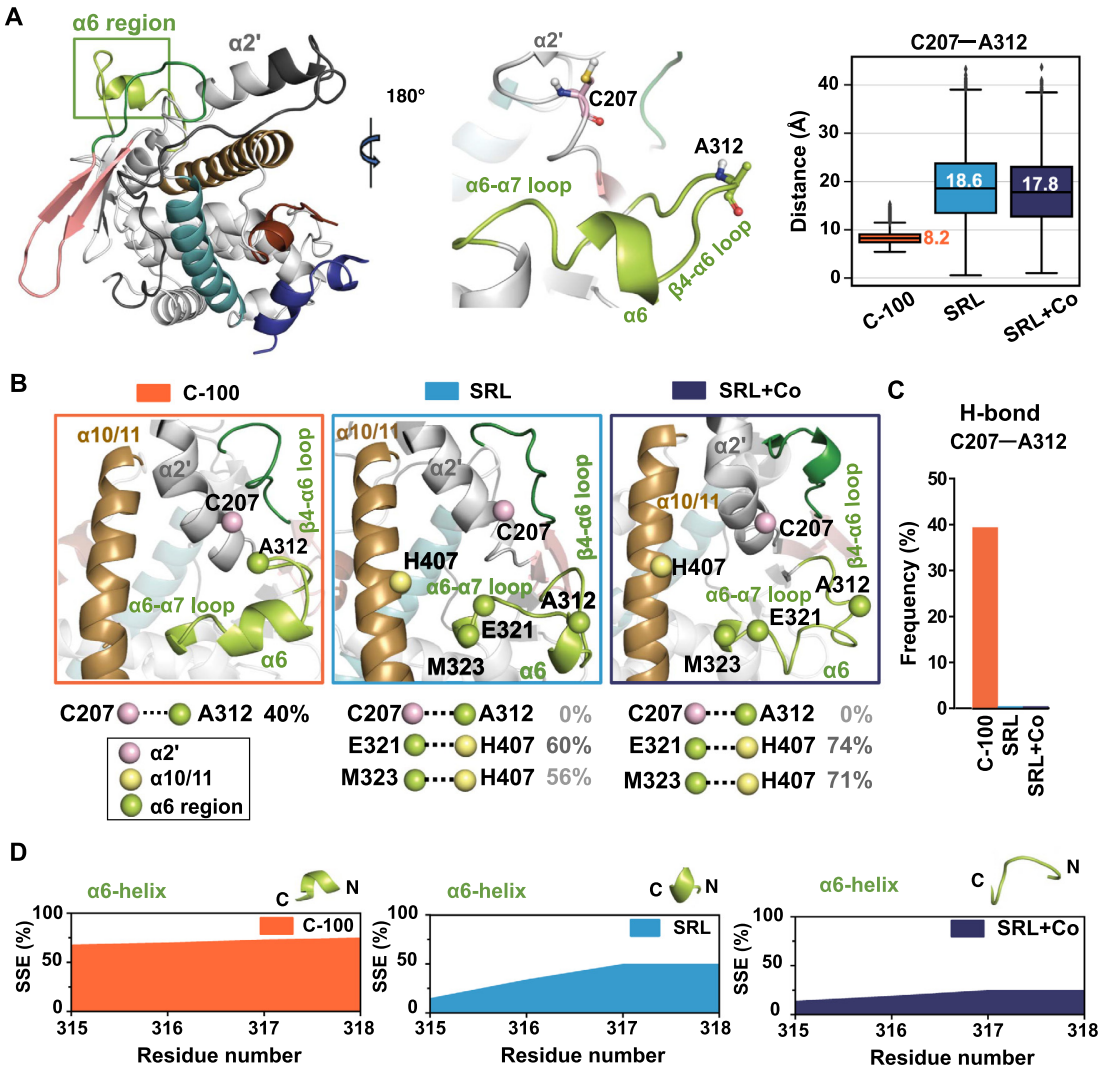
Compound 100 and SRL12813 induce distinct α6 region conformations. (A) Location of the α6 region in the PXR-LBD. Cα–Cα distance between C207 (of α2′) and A312 (of β4-α6 loop), which are shown in stick representation in the middle panel, illustrate that the compound 100 promotes a closed configuration of β4-α6 loop, while more open conformations are observed with the agonist. In the boxplots, box represents interquartile range (IQR: 25–75%); the black line represents the median (values are also displayed next to each box); shown in black vertical lines, whiskers represent the data within 1.5*IQR; outliers are indicated with diamond symbols. Distances were monitored each ns, i.e., there are: ∼30,000 individual datapoints for C-100; ∼20,000 for SRL; and ∼10,000 for SRL + Co. (B) Representative snapshots of the α6 region configuration of each system, water bridge and H-bond interaction differences. Closed configuration of β4-α6 loop in C-100 (left, orange box), opened configurations in agonist systems SRL (middle, light blue box) and SRL + Co (left, dark blue box). Selected water bridge interaction frequencies of the α6 region C-terminus residues (location of the Cα-atoms of each residue are indicated with spheres) are shown for each system (see also SI Fig S4). Spheres belonging to the same helix/region are coloured similarly. The colours of the helices are same as in Fig. 1A. C207–A312 interaction is H-bond and other interactions are water bridges. (C) C207–A312 H-bond interaction frequencies (0% for SRL and SRL + Co). (D) Secondary structure of α6 helix appears more stable with compound 100 than with SR12813. Area plots represent the observed secondary structure element (SSE) of the α6 helix in percentage throughout the simulation. Representative snapshots of the α6 secondary structure are displayed above related plot.

We next pursued for a more detailed analysis of these PCA-highlighted dynamic regions of PXR-LBD. First, we focused on the α6 region (residues 309–323), comprising β4-α6 loop (N-terminus of the region), α6 helix and α6-α7 loop (C-terminus of the region) (Fig. 3A). Movement of β4-α6 loop was associated to both PC1 and PC2, appearing even more extensive with PC2 (Fig. 2B, SI Movie M2). To inspect the conformation of this loop, we calculated the distance between A312 (located on β4-α6 loop) and C207 (located on C-terminus of α2′) (Fig. 3A). Clearly smaller distances are observed in the presence of compound 100 (median of 8.2 Å), whereas both agonist-bound systems display significantly longer distances between these two residues (medians of 17.8 Å and 18.6 Å for SRL + Co and SRL, respectively) (Fig. 3A). This indicates that the β4-α6 loop favours an open configuration with SR12813, where this loop resides far from α2′ (Fig. 3B). Conversely, a closed conformation is preferred with compound 100, where the β4-α6 loop is close to α2′. This closed conformation appears to be stabilized via a H-bond between A312 and C207, which is not observed with the agonist (Fig. 3C). Furthermore, we noted that the closed conformation of the β4-α6 loop appears to stabilize the secondary structure of α6 helix (Fig. 3D). While the helical configuration is dominated with compound 100, in agonist-bound systems this secondary structure is more unstable.

Motta et al. predicted that the water channel between α2 and α6 would be an entry pathway for SR12813 [40]. Therefore, we next investigated water-mediated interactions in these regions. Again, notable differences among the systems with these interactions appeared (Fig. 3B; SI Fig. S4). For instance, in C-terminus of α6 region, a water-bridged interaction between E321 and H407 (located on α10/11) is frequent in both agonist systems (60–74%), while it is relatively absent in C-100 (below 6%). Moreover, H407 forms a water-bridged interaction with M323 with agonist (56% and 71%, SRL and SRL + Co, respectively), which is again almost inexistent with the compound 100 (Fig. 3B). N-terminus of α6 region is connected to the C-terminus of α2′ helix (residue K210) with system specific water-bridged interactions (SRL and SRL + Co, E309; C-100, E309 and D310) (SI Fig. S4). Regardless of the differences observed for water bridged and H-bond interactions in α6 region, a comparable interaction profile among systems appears between D205 (located on α2′) and R410 or R413 (located on α10/11) (SI Fig. S4). Overall, the α6 region exhibits a ligand-dependent configuration, which is associated with specific intramolecular interactions within the PXR-LBD.

### 2.3 Different polar interactions contribute to stabilization of compound 100 and SR12813 in PXR-LBP

**Fig. 4:**
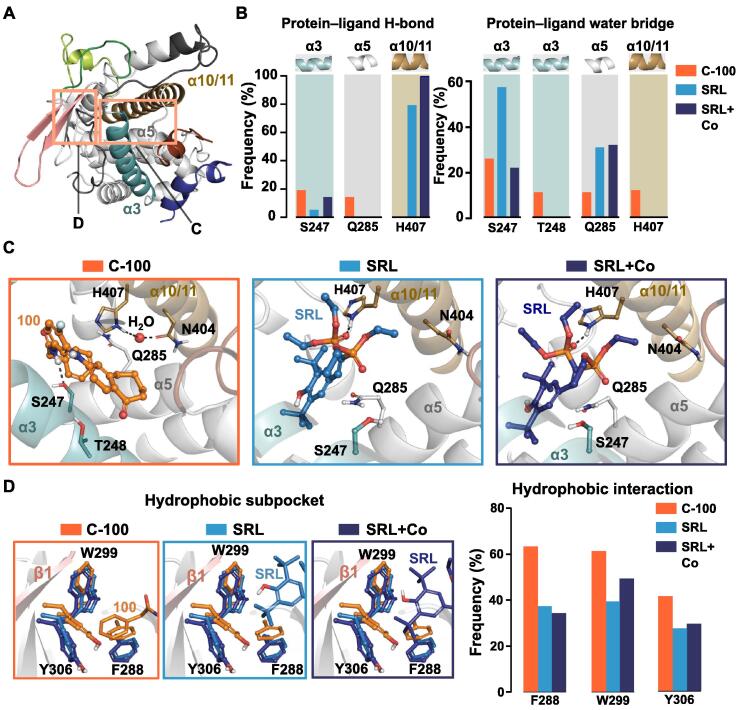
The key interactions between PXR-LBD and ligands. (A) Locations of the binding site and hydrophobic subpocket in the PXR-LBD, which are shown in more detail in C and D. (B) Protein–ligand H-bond and water-mediated interaction frequencies in individual systems. (C) Representative snapshots of LBP in C-100, SRL and SRL + Co systems. The secondary structure is coloured as in A. H-bonds are displayed with dashed black lines. Water molecule that forms bridge between H407 and N404 in C-100 is illustrated with a red sphere. (D) Superposition of hydrophobic subpocket of both agonist systems over C-100. Residues displayed in dark blue, SRL + Co; light blue, SRL; orange, C-100. Frequencies of hydrophobic interactions between ligands are illustrated in the bar plot. Hydrophobic interactions include: π − π interactions (face-to-face and face-to-edge) and hydrophobic interactions (see details in methods). (For interpretation of the references to colour in this figure legend, the reader is referred to the web version of this article.)

The analysis of α6 region interactions revealed that in the presence of compound 100, unlike with SR12813, H407 is not involved in water-mediated interactions between α6-α7 loop and α10/11. As H407 is one of the key residues of PXR-LBP commonly participating in interactions with ligands [[44], [45]], we next shifted our attention to protein–ligand interactions (Fig. 4). Based on ligand RMSF values, both ligands are relatively stable throughout the simulations (SI Fig. S5); thus, the data is suitable for comparing interactions between the ligands and among systems. While H407 displays a stable H-bond with SR12813 (∼80–100%), only a water-mediated interaction occurs with compound 100 (∼15%) (Fig. 4A–B). With compound 100, H407 prefers a conformation where it forms a water bridge (∼46%) to N404 (located on α10/11). This conformational preference shifts H407 away from the α6 region and beyond the reach of the compound 100 (Fig. 4C). Interestingly, our simulations display a stable H-bond interaction between N404 and G278 (on α4) with SR12813 (∼93–95%) and this interaction appears only with 23% frequency in C-100. This discrepancy could be the result of the N404 involvement in the water mediated interaction with H407. Compound 100 displays H-bond and water mediated interactions to S247 and Q285 from its amide (15–20%). From these residues, SR12813 exhibits a direct H-bond interaction only with S247. The missing H-bond to Q285 is compensated with water-mediated interactions. Similarly, increased frequency of water-mediated interaction (58%) appears in SRL, where diminished H-bond interaction to S247 exists (Fig. 4B). Furthermore, compound 100 displays an additional water-mediated interaction to T248 — albeit with low frequency (∼10%) — from its NH linker between benzosuberone and fluorophenyl. Overall, limited polar interactions are observed for both ligands, with a clear difference in their H407 interactions.

### 2.4 Compound 100 and SR12813 share similar hydrophobic interactions, except in αAF-2 region

The most important interaction region for different PXR ligands is the hydrophobic subpocket, which is formed by a triad of hydrophobic residues: F288, W299 and Y306 [45]. Here, we observed a similar protein-ligand interaction pattern in the presence of SR12813 (with and without coactivator) or compound 100 (Fig. 4D). SR12813 forms hydrophobic interactions to F288 and Y306 for about 30–40% and to W299 for ∼40–50% in both systems. Similarly, compound 100 displays hydrophobic interactions between benzyl group and F288, W299 (60%) and Y306 (approximately 45%). The slightly increased frequency of hydrophobic interactions for compound 100 could be attributed to the structural characteristics of the compounds. Compound 100 does not contain any polar atoms in its terminal group that binds to the hydrophobic subpocket, while SR12813 contains an aromatic hydroxyl group in its structure binding to this region which can explain the observed shift of the Y306 and F288 side chain in C-100 compared to agonist systems (Fig. 4D; SI Fig. S6).

Along with the hydrophobic subpocket, other regions of LBP also contribute to hydrophobic interactions with the ligands (SI Fig. S7A). Namely, α3, α5, α10/11 and the loop connecting α11 to αAF-2 contain hydrophobic residues that displayed hydrophobic interactions. Clearly higher interaction frequencies with F420 and F429 (located on αAF-2) are observed for SR12813 (20–45%), while compound 100 displays 0–10% interaction frequencies with these residues. Moreover, compound 100 exhibits hydrophobic interaction with F251 (located on α3-helix), which is not present with SR12813.

Collectively, both ligands show similar hydrophobic interaction patterns within the PXR-LBP, with the exceptions of F251 (located on α5) and αAF-2 residues F420 and F429. Since F420 is located on the loop connecting α11 to αAF-2 and this loop has a critical role in the αAF-2 localization [[46], [47]], we explored F420 interactions to its neighbouring residues (SI Fig. S7B). With SR12813, F420 displays hydrophobic interactions to L411 (located on α11) for roughly 28–40% in both agonist systems. This interaction is infrequent with compound 100 (∼8%). F420 also interacts with I414 for 18–30% in both agonist systems, while diminished interactions (only ∼3%) appear with compound 100. Interestingly, RMSF of F420 is higher (2.4 Å) with C-100 than with SRL (1.2 Å), demonstrating a high flexibility of this amino acid residue with compound 100.

### 2.5 αAF-2 is stabilized with SRL12813 and destabilized with compound 100

Compound 100 exhibited diminished interactions with αAF-2. All known ligand-dependent nuclear receptors require αAF-2 domain for an effective interaction with a coactivator [48]. This domain plays a crucial role in the formation of a suitable platform for the coactivator binding on the LBD surface. Therefore, we next investigated more closely the behaviour of αAF-2 in different systems.

**Fig. 5:**
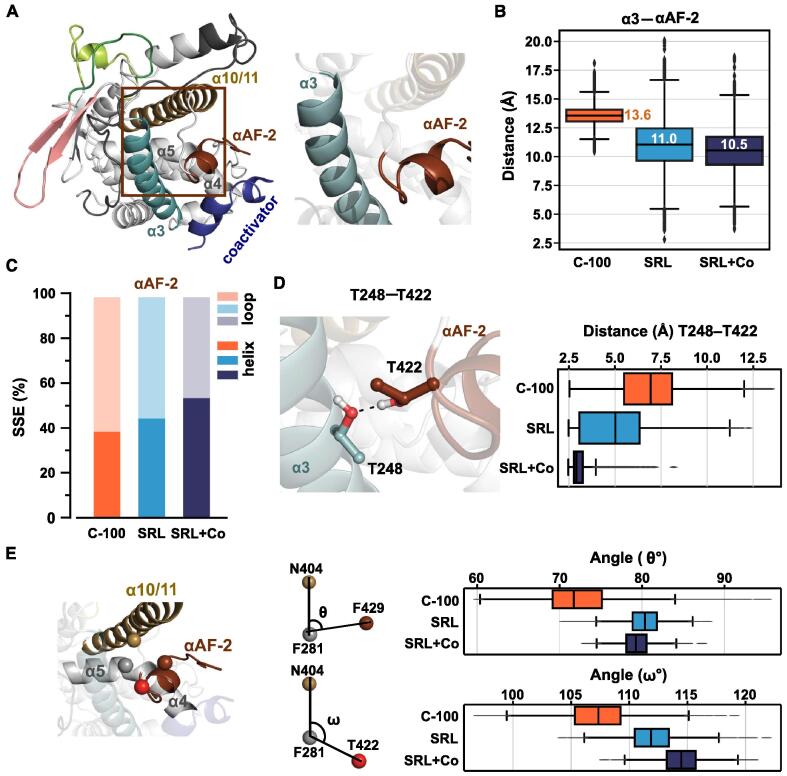
Destabilization of αAF-2 with compound 100. (A) PXR-LBD crystal structure and close view of α3-helix and αAF-2. (B) Box plot represents the distribution of distance between centre of mass of α3-helix (residues 240–260) and αAF-2 (residues 423–430). (C) Secondary structure element (SSE) of αAF-2 throughout the simulation. Proportion of helix shown in darker colour, loop-like structure shown in lighter colour. (D) H-bond interacting residues T248 and T422 (PDB ID: 1NRL [22]). Distance between sidechain oxygen atoms of T248 and T422 are shown in box plot. (E) θ and ω define the angle consisting of N404, F281, F429 and N404, F281, T422, respectively where F281 is the apex. Box plots display the distribution of these angles among the three systems.

First, we monitored the distance between αAF-2 and α3-helix (Fig. 5A–B). As α3-helix (residues 240–260) is stable in all simulations (RMSF < 1 Å), this distance enables the assessment of the relative position of αAF-2 to the LBD. In the presence of compound 100, this distance is increased (median of 13.6 Å) compared to what is observed for SR12813 (medians ∼11 Å). Furthermore, we noticed that the unfolded loop-like conformation of αAF-2 is slightly preferred with compound 100, in comparison to SR12813 (Fig. 5C). The presence of the coactivator further stabilizes the alpha-helical secondary structure of αAF-2 based on SRL + Co. Based on the crystal structure of PXR-LBD–SR12813 with coactivator, a H-bond interaction of T248 and T422 stabilizes αAF-2 closer to α3-helix (Fig. 5D). In the simulations, distance between the hydroxyl-oxygens of these residues is increased with compound 100 (median of 7.2 Å) (Fig. 5D), decreasing the H-bond frequency between these residues to 5%. In SRL + Co system, the median value of this distance is 2.9 Å and H-bond contact appears with approximately 80% frequency. Without the coactivator, SR12813 displays values between these two systems (median of 5.4 Å, H-bond frequency 30%). Finally, we analysed the spatial orientation of αAF-2 relative to LBP using angle calculations (Fig. 5E). To this end, we selected F281 (located on α5) as the apex (see details of angle selection in methods). The angle between N404 and F429 vectors (θ) is the smallest in the presence of compound 100 (median of ∼71°), while larger values are observed for SRL12813 (∼79–80°). This trend applies with the angle between N404 and T422 vectors (ω), where the smallest angle appears with compound 100 (∼107°) and agonist systems display larger angles (∼112–115°). Overall, the configuration of αAF-2 is affected by the bound ligand, and compound 100 appears to destabilize the LBD surface associated active conformation.

To evaluate the impact of the SRC-1 in the stabilization of the αAF-2 region with compound 100, we established an arbitrary system including PXR-LBD, compound 100 and SRC-1 (C-100 + Co; total simulation time of 10 µs). Also in this system, the configuration of αAF-2 is destabilized by compound 100 and its behaviour reflects that of C-100 (SI Fig. S8). The observed distance between αAF-2 and α3-helix with C-100 + Co (median of 13.7 Å) is not far from C-100. Furthermore, the distance between the hydroxyl-oxygens of T248 and T422 (median of 6.9 Å) is close to the C-100 than that of SRL and SRL-Co. Regarding the spatial orientation of the αAF-2 relative to LBP, the angles θ and ω of C-100 + Co fall in somewhere between C-100 and SRL systems. Finally, increased RMSF values of SRC-1 in C-100 + Co compared to SRL + Co, demonstrate the coactivator instability with compound 100. Therefore, even in the presence of SRC-1, compound 100 appears to destabilize the PXR-LBD surface on the αAF-2 region, encumbering the stable binding of SRC-1. This MD simulation data agrees with the experimental data that demonstrates the failure of compound 100 to recruit SRC-1 to PXR-LBD in coactivator recruitment mammalian 2-hybrid assay [42].

### 2.6 Markov state modelling reveals compound 100 specific PXR-LBD conformations

**Fig. 6:**
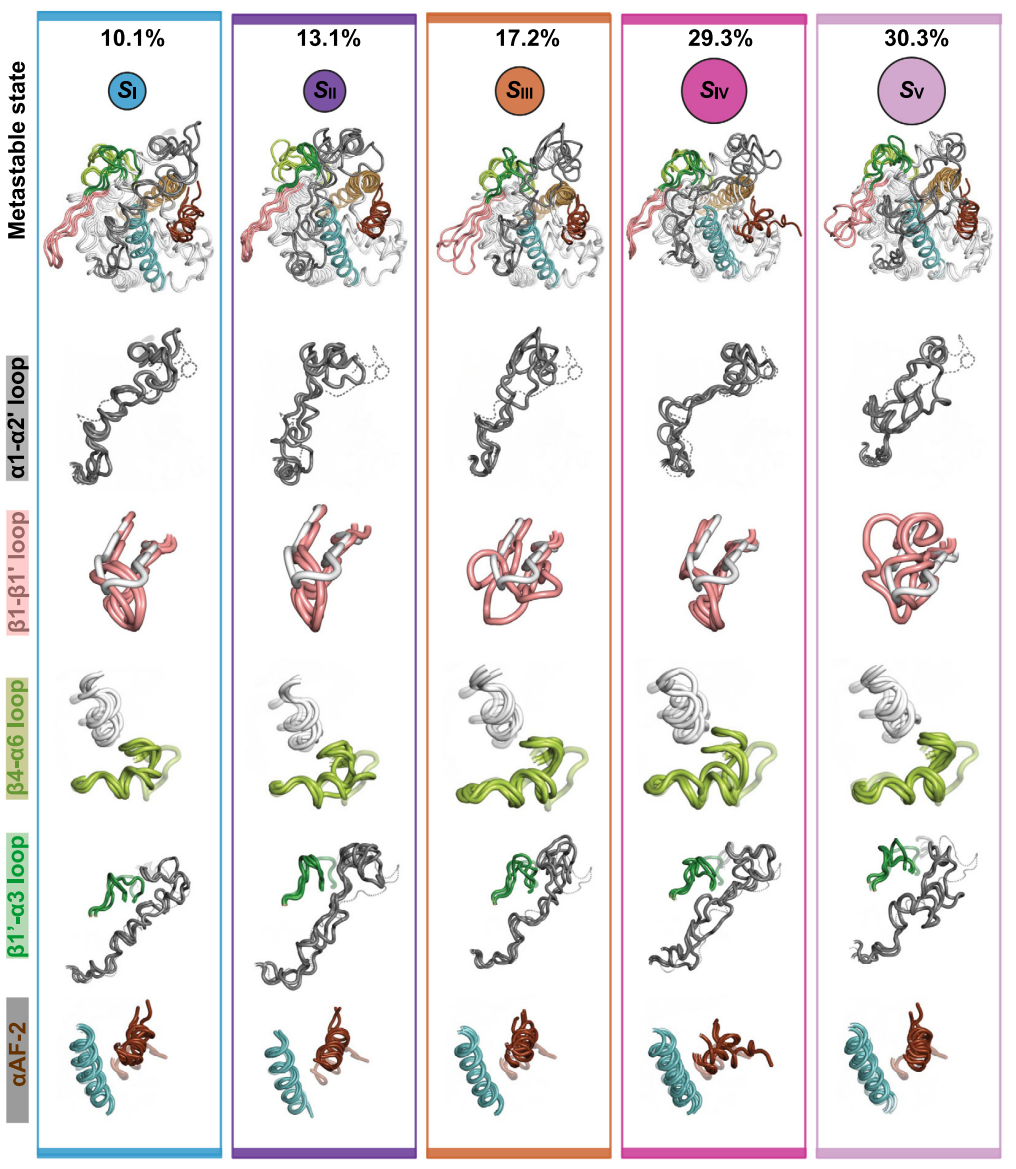
Markov state modelling reveals five metastable states for the PXR-LBD bound to compound 100. The five metastable states (*S*_I–V_) are displayed with three structures. The area of a circle is proportional to the equilibrium probability (π_i_) associated to each metastable state (also shown with %). The selected substructure configurations are shown individually, with a reference conformation from an agonist bound crystal structure (PDB ID: 1NRL). Reference crystal substructures are illustrated as follows: α1-α2′ loop, grey dashed line (disordered in the crystal structure); β-β1′ loop, white; β4-α6 loop, transparent light green, located in the vicinity of α2′ (white helix); β1′-α3 loop, transparent dark green; αAF-2, transparent dark brown, located in the vicinity of α3-helix (cyan helix). The structures of the metastable states are also provided in supplementary PyMOL (v.2.4.2) session-files. (For interpretation of the references to colour in this figure legend, the reader is referred to the web version of this article.)

We next aimed for a deeper understanding of PXR-LBD conformational dynamics when in complex with compound 100. To this end, we conducted Markov state modelling (MSM) approach that enables the study of long timescale statistical dynamics of a protein by identifying relevant kinetic states (metastable states) and the probability distribution among these states [[49], [50]]. MSM identified five metastable states (*S*_I–V_) for the PXR-LBD bound to compound 100 (Fig. 6; SI Fig. S9). The two most dominant metastable states, *S*_IV_ and *S*_V_, appear with ∼29% and ∼30% equilibrium probabilities, respectively. Other states (*S*_I_, *S*_II_, *S*_III_) display lower probabilities in the range of 10–17%. Overall, conformations of LBD subregions in metastable state derived structures are distinct from the agonist associated. Moreover, specific conformations are preferred in individual metastable states. For the flexible α1-α2′ loop, which flexibility is reduced by compound 100 compared to SR12813 (Fig. 2C), a state-specific conformation appears in *S*_I_ and *S*_IV_, while in *S*_II,_ *S*_III_ and *S*_V_ there exist no clear configuration for this loop (Fig. 6). Of note, in the agonist-bound reference crystal structure this loop is disordered (SI Fig. S10). The β1-β1′ loop in *S*_I_, *S*_II_, *S*_IV_ (altogether ∼53%) adopts a clearly defined folded conformation, where it is tightly packed on the LBD (Fig. 6). Configuration of this region is more ambiguous in *S*_III_ and *S*_V_. In the agonist bound crystal structure, β1-β1′ loop appears in an extended conformation, more distant from the LBD (Fig. 6; SI Fig. S10). Indeed, the distance between D219 (apex of β1-β1′ loop) and α3-helix is smaller with compound 100 than with SR12813 bound systems (SI Fig. S11A). For the β4-α6 loop, almost identical conformation is represented in all states, where it resides close to the α2′ (residue C207) (Fig. 6). Conversely, an open configuration for this loop is observed in the agonist-bound crystal structure (Fig. 6, SI Fig. S10). Again, distance between α3-helix and A312 is smaller in C-100 compared to SRL and SRL + Co (SI Fig. S11B). The β1′-α3 loop in *S*_II_, *S*_IV_ and *S*_V_ (altogether ∼73%) deviates the most from the agonist-associated conformation, while in *S*_I_ and *S*_III_ this deviation is not that evident (Fig. 6). Interestingly, the αAF-2 region appears in a quite well-defined conformation in *S*_I_, *S*_II_, *S*_III_ and *S*_V_, (altogether ∼71%), where it is shifted away from the α3-helix (Fig. 6). Moreover, in *S*_IV_ αAF-2 appears with a more disordered conformation. These αAF-2 configurations agree with the calculated αAF-2 related distances and angles (Fig. 5). Altogether, MSM revealed unique conformations for the PXR-LBD bound to compound 100 in four regions, β1′-α3 loop, β1-β’loop, β4-α6 loop and αAF-2, that are distinct from agonist-associated conformations.

### 2.7 Mutations provide insights to the PXR activation

**Fig. 7:**
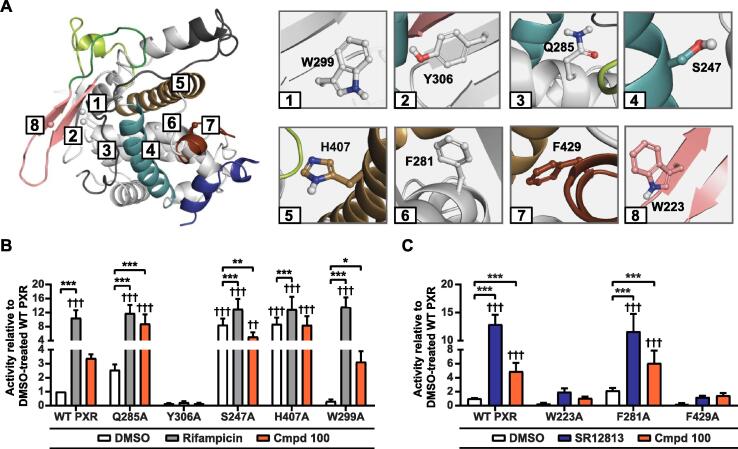
Ligand-induced effects on activation of PXR mutants. (A) The reference crystal structure (PDB ID: 1NRL) illustrating the locations of the generated PXR mutants (locations of the Cα-atoms of each mutated residue are indicated with spheres). Each number represents the location of a mutated residue (left). Close-ups of the mutated wild type residues are shown in stick representation (right). (B) Observed ligand induced PXR activation in reporter gene assay. Transfected cells were treated 24 h with 0.1% DMSO, 10 µM rifampicin or 1 µM SR12813 (C), or 10 µM compound 100. Data is presented as mean relative activity ± SD to DMSO-treated WT PXR from five independent experiments with technical triplicates. †††p < 0.001 compared to DMSO-treated WT PXR analysed with two-way anova with Dunnett‘s multiple comparisons test. *p < 0.05, **p < 0.01, ***p < 0.001, compared to DMSO-treated respective mutant analysed with two-way anova with Tukey‘s multiple comparison test.

We next evaluated experimentally the effect of eight different mutations on PXR activity and its ligand-induced activation (Fig. 7A). The alanine mutations involved selected LBD key residues with diverse locations around PXR-LBD. The competitive antagonist compound 100 induces moderately the reporter gene CYP3A4 expression with WT PXR at the tested concentration, while with the full agonists, rifampicin and SR12813, the inducement of the gene expression is manyfold (for more detailed biological characterisation of compound 100 see [42]). Mutations of the hydrophobic subpocket forming residues W299 and Y306 resulted in distinct outcomes. While W299A retains the inducibility by rifampicin and behaves similarly as wild type, Y306A renders PXR inactive without any ligand-inducibility (Fig. 7B). From the mutations of the polar residues participating in H-bond interactions (Q285, S247 and H407), S247A and H407A increased the basal level of PXR activity, transforming it into a constitutive active form. These mutations appear still inducible by the PXR agonist rifampicin, and compound 100 suppresses the activity of S247A. MD simulations displayed hydrophobic interaction between F281 and both ligands (Fig. S5). Nevertheless, F281A did not alter PXR activity or inducibility by the ligands. F429A, located in αAF-2, rendered PXR inactive, and was not inducible by the ligands. Finally, W223A mutation in the putative PXR homodimerization interface [51], also resulted in the loss of PXR activity. Overall, mutation analysis revealed the important role of the key residues in modulating PXR activity and activation.

### 2.8 Hydrophobic subpocket binding moiety does not explain PXR conformational behaviour associated to compound 100

**Fig. 8:**
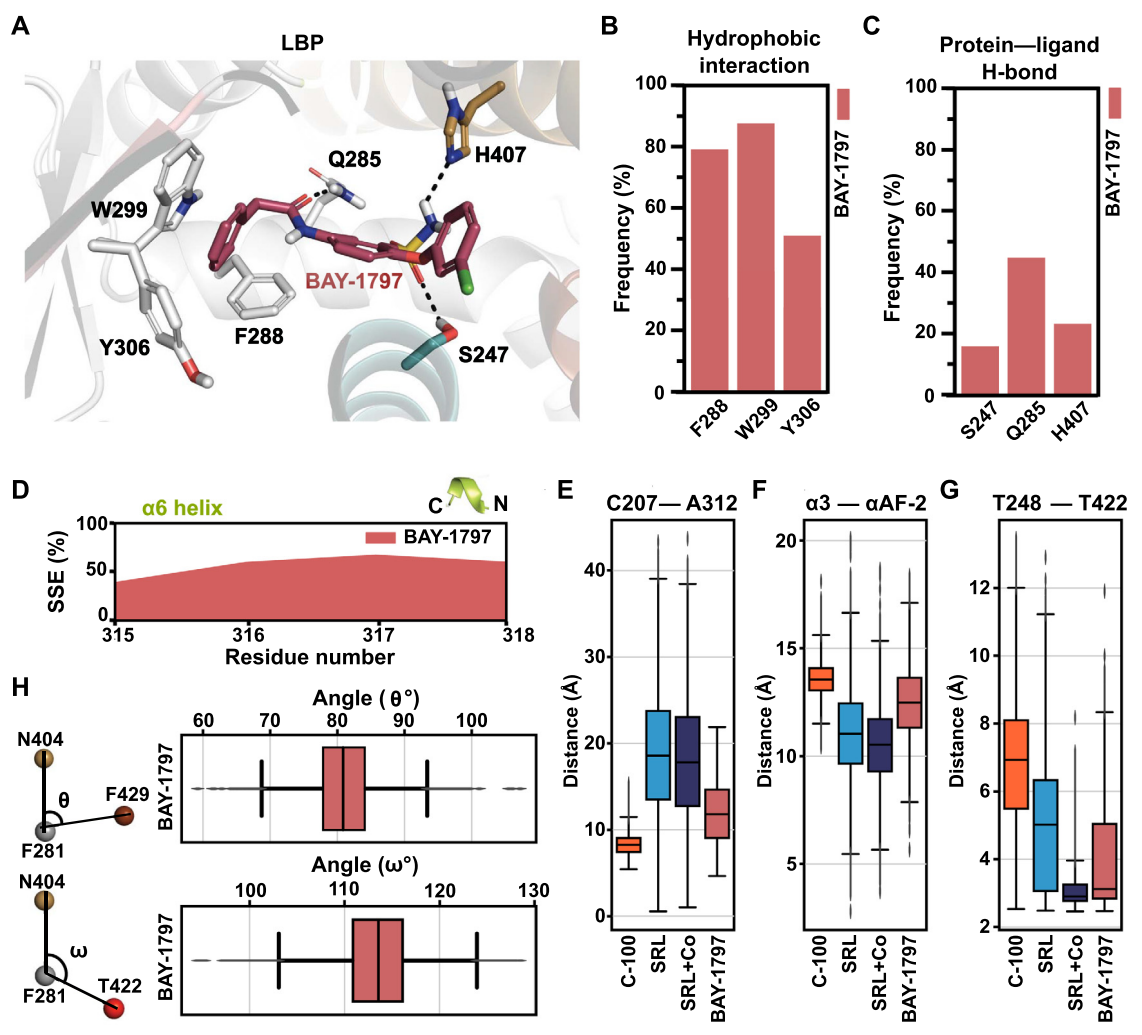
PXR-LBD–BAY-1797 simulations display behaviour of an agonist. (A) A representative snapshot of LBP with BAY-1797. The colour of the residues is as in PXR-LBP in Fig. 1B. H-bonds are displayed with dashed black lines. (B) Protein–ligand hydrophobic interactions frequencies between ligand and protein in individual systems are illustrated in the bar plot. Hydrophobic interactions include: π − π interactions (face-to-face and face-to-edge) and hydrophobic interactions (see details in methods). (C) Protein–ligand H-bond interactions frequencies between ligand and protein in individual systems are illustrated in the bar. (D) Area plots represent the observed secondary structure element (SSE) of the α6 helix in percentage of BAY-1797 throughout the simulation. (E) Cα–Cα distance between C207 (of α2′) and A312 (of β4-α6 loop) illustrates that the BAY-1797 promotes a further closed configuration of β4-α6 loop like, while more open conformations are observed with the agonist. (F) Box plot represents the distribution of distance between centre of mass of α3-helix (residues 240–260) and αAF-2 (residues 423–434). (G) Distance between sidechain oxygen atoms of T248 and T422 are shown in box plot. (H) Internal protein interactions of αAF-2 region. θ and ω define the angle consists of N404, F281, F429 and N404, F281, T422, respectively where F281 is the apex. Box plots display the distribution of these angles with BAY-1797 (median of ∼80° for θ and ∼113° for ω). Respective medians for the other systems are for θ: C-100, ∼71°; SRL, ∼80°; SRL + Co ∼79° and ω: C-100, ∼107°; SRL, ∼112°; SRL + Co ∼115°.

Werner et al. reported a P2X4 inhibitor BAY-1797, which also activated PXR with a minimum efficacious concentration of 1.7 μM [43]. We got interested in BAY-1797, as it shares an identical phenylacetamide moiety with compound 100 (Fig. 1C). To investigate and compare the behaviour of BAY-1797 with compound 100, we carried out 10 µs MD simulations for PXR-LBD–BAY-1797. As a starting configuration for these simulations, we utilized a crystal structure of a close analogue of BAY-1797, which exhibits one additional methyl group compared to BAY-1797 (PDB ID: 6HTY [43]). The overall dynamical behaviour of the protein in the simulations (RMSF) follows a similar trend with B-factors of the crystal structure. (SI Fig. S3C). BAY-1797 is well accommodated in the PXR-LBP and its aromatic benzyl group is oriented into the hydrophobic subpocket (Fig. 8A). A comparable profile with compound 100 is observed for BAY-1797 in π − π interactions to W299, F288 and Y306 (Fig. 8A and B, Fig. 4D). In contrast to compound 100, however, BAY-1797 displays a stable H-bond to Q285 and interaction to H407 (43%), which is closer to the SR12813 interaction profile (Fig. 8C). The secondary structure stability of α6-helix with BAY-1797 resembles compound 100 (Fig. 8D). Nevertheless, based on the distance of A312–C207 (located on β4-α6 loop and on C-terminus of α2′, respectively) BAY-1797 falls somewhere in between of SRL12813 and compound 100 (Fig. 8E), indicating unique conformation for this region. In addition, distance between αAF-2 and α3-helix in PXR-LBD–BAY-1797 appears in between the SR12813 and compound 100 systems (Fig. 8F). However, the distance between hydroxyl-oxygens of T248 and T422 suggests that with BAY-1797, αAF-2 can acquire a stable active configuration that is required for PXR activation (Fig. 8G). This leads hydrophobic interaction with F420 for 30% comparable to SR12813 but no interaction was observed with F251 and F429 in presence of BAY-1797 (SI Fig. S12). The angle between N404 and F429 vectors (θ) is larger (median of ∼80°) compared to that of compound 100 and close to what is observed for SR12813 (Fig. 8H; Fig. 5). This trend applies with the other angle between N404 and T422 vectors (ω), with an angle (median of 114°) close to that of SR12813. Overall, the configuration of αAF-2 appears to be stabilized on LBD surface with BAY-1797.

## 3 Discussion

Since the mechanism of PXR antagonism has not been elucidated and X-ray crystallography is unable to capture the ligand-induced conformational dynamics of PXR-LBD [13], we utilized here in silico approach to disclose the putative ligand-dependent differences in conformational dynamics of PXR-LBD. Our MD data suggest ligand-dependent discrepancy in the conformational preference on different LBD regions. This discrepancy is observed in α6 region, αAF-2, α1-α2′, β1′-α3 and β1-β1′ loop.

Earlier studies reported that α6 is less folded or very dynamic in PXR [[52], [53]], enabling a flexible cavity to accommodate ligands of different sizes [19]. We found that compound 100 induced a tightly packed and folded conformation in this region, while a looser configuration was preferred with SR12813. Motta et al. predicted that the water channel between α2 and α6 would be an entry pathway for SR12813 [40]. This region appears as a favourable ligand pathway among nuclear receptors [54]. Interestingly, our simulations suggest that water bridged interactions mediated by E321 and M323 on a6 and H407 on α10/11 appear on this site with SR12813, where no such interaction exists with compound 100. A common feature of a PXR agonist is to engage in direct interactions with H407 of the α10/11 [[25], [44], [45], [55]], and H407A mutation renders PXR constitutively active. Our simulations suggest that compound 100 does not rely on interactions with H407. This appears to be related to the engagement of H407 in water mediated interaction to N404.

Anami et al. [46] proposed a model for vitamin D receptor (VDR) activation or repression called “folding-door model” to explain VDR-LBD activity. In this model, α11 cooperates with αAF-2 in a way that in the presence of an agonist αAF-2 is stabilized close to α3-helix, forming internal interactions with the α11 kink. These interactions close the door (α11). Meanwhile this kink is open in the presence of an antagonist and the α11-αAF-2 loop plays an important role in the unsuitable αAF-2 positioning for receptor activation. Here, our simulations display a stable H-bond interaction between N404 (on α11) and G278 (on α4) with SR12813, which is an infrequent interaction with compound 100. Hence, we hypothesize that with PXR the ligand-dependent N404 conformation – and the lack of water bridge interaction between H407 and α6 in presence of compound 100 – may result in a more flexible α11 with compound 100. This would result in the rearrangement of the flexible α11-αAF-2 loop. We also observed a ligand-dependent discrepancy in F420 and α11 interactions, and diminished hydrophobic interaction between compound 100 and this loop. Our findings agree with earlier results reported by Shizu et al. that revealed the important role of F420 (on α11-αAF-2 loop) in PXR αAF-2 stabilization [47]. Moreover, mutation of F429A, located in αAF-2, rendered PXR inactive, and was not inducible by the ligands highlighting its relevance in the binding. Altogether, we conclude that in presence of compound 100 these motifs play a role in dislocation of αAF-2 from the vicinity of α3-helix, which is demonstrated by the distance and angle calculations of αAF-2 as well as by the MSM. This dislocation of αAF-2 was also observed in the simulations of compound 100 in the presence of SRC-1 (C-100 + Co). With SR12813 a stable conformation of αAF-2 is maintained, providing a suitable platform for the co-activator binding and subsequent PXR activation. Of note, not only is compound 100 impairing binding of coactivator SRC-1 but also binding of corepressor SMRT [42]. This is in contrast to what is observed with the full antagonist SPA70, which enables recruitment of the corepressor [27]. Therefore, the induced conformational changes by compound 100 enable competitive PXR antagonism but not full antagonism, which may require that the ligand induces suitable conformations for the corepressor binding.

The adaptability of the hydrophobic subpocket in PXR is emphasized by the different conformations of Y306 and F288 that exist with SR12813 and compound 100. Meanwhile, mutation of Y306 renders PXR inactive with both ligands. This could be attributed to the loss of PXR-LBD integrity, as in multiple PXR crystal structures there exists a H-bond interaction between Y306 and H242 (located on α3-helix), highlighting the important role of this residue in PXR structural stability.

The highest flexibility of PXR-LBD was observed in α1-α2′ loop with both compound 100 and SR12813. This flexibility is well exemplified by structural data, as this region is disordered in all publicly available PXR structures. Earlier studies show also the high degree of flexibility in this region in the presence of agonist [[56], [57]]. Our MD data displayed somewhat lower RMSF for this region with compound 100. MSM suggested that in some metastable states there exists rather state-specific conformation for this loop, while in others states more disordered configuration appears. It is worth noting that this loop (in proximity of α2′) is part of one of the proposed water channels in PXR [40] that stretches along β1′-α3 loop [58]. For this β1′-α3 loop, MSM displayed a clear deviation from the agonist-associated conformation. Further study is required to disclose the role of different conformations of the ambiguous α1-α2′ loop together with β1′-α3 loop, to better understand their interrelation on the ligand binding and specificity.

Our simulations suggest that the β1-β1′ loop is tightly packed on the LBD with compound 100. Conversely, with SR12813 this substructural element resides farther from the α3-helix. Interestingly, the W223A mutation on this outer interface renders PXR inactive with both SR12813 and compound 100. The functional relevance of this residue was also revealed by Nobel et al. [51]. Of note, this residue is part of PXR-LBD insertion [58].

For BAY-1797, that shares an identical phenylacetamide moiety with compound 100, we observed an intermediate profile in between SR12813 and compound 100. It must be noted that SR12813 is a more potent PXR agonist than BAY-1797. In the hydrophobic subpocket BAY-1797 shows a similar behaviour as compound 100. In addition, in the presence of BAY-1797 the distance between αAF-2 and α3-helix is increased compared to SR12813, but not to the extent of compound 100. Nevertheless, the distance between T248 and T422 with BAY-1797 is close to what is observed with SR12813. It was noted by Werner et al. [43] that changing the 3-chlorophenoxy with a larger and more polar substituent alleviated PXR agonism, which could occur either by diminishing PXR binding or by disrupting the αAF-2. In this regard, we suggest that along with the classical mechanism of action of a nuclear receptor, the more specific distance of T248 and T422 may be useful when analysing potential risk for PXR agonism by MD simulations.

Overall, our results revealed the adaptability and ligand-specificity in PXR conformational behaviour. More folded β1-β1′ loop, compact α6 region, lower flexible α1-α2′ loop, dislocated β1′-α3 loop and αAF-2 are associated with compound 100. Our study provides more in depth understanding of ligand-induced changes in PXR-LBD substructures. Although our compound is a competitive antagonist [42] and not a full antagonist as SPA70 [27], these results still provide a putative template for designing PXR antagonists. Finally, the identified structural key-regions provide guidance how to potentially avoid PXR agonism.

## 4 Material and methods

### 4.1 MD simulations

Modelling was conducted with Maestro (Schrödinger Release 2019-4: Maestro, Schrödinger, LLC, New York, NY, 2019), and OPLS3e force field [[59], [60]], unless otherwise stated. For the simulations of C-100 and C-100 + SRC, we used the PXR crystal structure PDB ID: 4J5W (chain A) [61] and for the simulation of SRL and SRL + Co, we used the PXR crystal structure PDB ID: 1NRL (chain A) [22]. For BAY-1797 simulations, we applied its close analogue PXR co-crystal structure PDB ID: 6HTY (chain B) [43], and the redundant methyl group of the analogue was deleted. The residues missing in the C-terminal (G433 and/or S434) were added to the structures with Maestro tools. For SRL and C-100 systems, SRC-1 was removed. The proteins were prepared using Protein Preparation Wizard (Schrödinger LLC, New York, NY, 2019) [62]. Missing hydrogen atoms were added, bond orders were assigned using CCD database, missing side chains and loops (α1-α2 loop; residues 178–192 for 4J5W and α1-α2 loop; residues 178–191 for 1NRL and 6HTY) were filled to the structure using Prime [63], protonation states of amino acids were optimized with PROPKA (Schrödinger, LLC, New York, NY, 2019), the coactivator peptide was capped in both termini and the structures were minimized. To obtain the starting configuration for compound 100, Glide docking was conducted (Glide v. 7.7) [[64], [65]]. Before docking, compound 100, was prepared with LigPrep (Schrödinger, LLC, New York, NY, 2019) to assign the protonation state (Epik; at pH 7.0 +/-2.0) and the partial charges. For the docking of compound 100, default settings were applied, with residues H407, Q285, S247, H327 and F429 selected to define the active site, and the docking was conducted using the standard-precision (SP) and extra-precision (XP) level of accuracy [66]. The docking resulted in an U-shape pose (mainly accommodated in hydrophobic subpocket) from the SP (docking score: −10.542; glide emodel: −86.922) and an extended pose (oriented from hydrophobic subpocket with benzyl moiety, while benzosuberone oriented towards αAF-2 region) from XP (docking score: −13.647, emodel: −98.455). We evaluated these two distinguishable poses in short MD simulations (data not shown) and the pose with extended conformation displayed better stability during the simulation, which was selected as a starting configuration for compound 100 in the production simulations. Of note, based on our later evaluation by the QM Conformer predictor tool, this extended conformation is also proposed for compound 100 as the lowest energy conformation in water [42]. The same pose was also used in C-100 + Co simulation where the SRC-1 peptide was maintained.

For the simulations, we used Desmond MD simulation engine [67]. The prepared systems were solvated in a cubic box with the size of the box set as 15 Å minimum distance from the box edges to any atom of the protein. TIP3P water model [68] was used to describe the solvent and the net charge was neutralized using K^+^ ion with final salt concentration of 150 mM. RESPA integrator timesteps of 2 fs for bonded and near, and 6 fs for far were applied. The short-range coulombic interactions were treated using a cut-off value of 9.0 Å. Before the production simulations the systems were relaxed using the default Desmond relaxation protocol. Simulations were run in NPT ensemble, with temperature of 310 K (Nosé-Hoover thermostat) and pressure of 1.01325 bar (Martyna-Tobias-Klein barostat). For each system, simulations of five replicas with different lengths (2–4 µs) were carried out, resulting in total of 30 µs simulation data for C-100, 20 µs for SRL, 10 µs for SRL + Co, 10 µs for C-100 + SRC and 10 µs for BAY-1797 (SI Table 3; SI Fig. S13). With C-100, additional independent replicas derived from the original simulations were run to obtain sufficient sampling for MSM.

### 4.2 Analysis of MD simulation data

Principal component analysis. PCA was conducted for the backbone atoms using GROMACS tools (version 2019) (gmx covar and gmx anaeig) [69]. For GROMACS analysis, the Desmond trajectories were aligned and transformed to xtc-format, keeping only backbone atoms. Figures describing the extreme motions were generated and visualized using PyMOL-script Modevectors [70].

RMSD, RMSF, protein secondary structure elements (SSE) and interaction analysis. Maestro simulation interaction analysis tool (Schrödinger, LLC) was used for these analyses. For interaction criteria, default values were used. H-bonds: cut-off of 2.5 Å for donor and acceptor atoms, donor angle of 120° and acceptor angle of 90°. Hydrophobic interactions: cut-off of 3.6 Å between ligand's aromatic or aliphatic carbons and a hydrophobic side chain, π-π interaction was defined as two aromatic groups stacked face-to-face or face-to-edge. Water bridge interactions: default cut-off of 2.8 Å for donor and acceptor atoms, donor angle of 110° and acceptor angle of 90°.

Angle and distance calculations. Maestro event analysis tool (Schrödinger, LLC) was used. Distances between specific secondary structure elements were calculated using their centers of mass with the Maestro script *trj_asl_distance.py* (Schrödinger LLC). For α3-helix residues 240–260 and αAF-2 residues 423–430 were used. For the distance calculation Cα atom of each residue was used. The angles were calculated using the Cα atom of N404, F281, F429 for θ and Cα atom of N404, F281, T422 for ω with the Maestro script *event_analysis.py* and *analyze_simulation.py* (Schrödinger LLC).

Markov state modelling. Bayesian MSM was generated with PyEMMA 2 following the general recommendations [71]. As an input, we used full protein backbone torsion angles. Time-lagged independent component analysis (TICA) was used for dimension reduction [72], using 10 ns as a lag time, and two dimensions. The output of TICA was discretized to microstates using the k-means clustering (number of clusters set as √N), and the microstates were assigned in five macrostates (metastable states) by the Perron-cluster cluster analysis (PCCA++) method [73]. Implied timescales and Chapman-Kolmogorov test suggest a valid model (SI Fig. S14).

Structure and data visualization. Structure visualization was conducted with PyMOL v.2.4 (Schrödinger LLC, New York, NY, USA). Data visualization was completed by Python 3.7, seaborn [74], matplotlib [75] and GraphPad prism (v. 8.0.0 for Windows, GraphPad Software, San Diego, CA, USA).

### 4.3 Chemicals and reagents

DMSO was purchased from Sigma Aldrich (Taufkirchen, Germany). Rifampicin and SR12813 were obtained from Tocris Bioscience (Bristol, UK). Compound 100 was synthesized in house [42]. Minimum essential medium (MEM) and Trypsin-EDTA were purchased from Thermo Fischer Scientific (Waltham, MA, USA). L–glutamine and penicillin-streptomycin mixture were provided by Biozym (Hessisch Oldendorf, Germany). Fetal bovine serum (FBS) was obtained from Biowest (Nuaillé, France).

### 4.4 Plasmids

Full-length human PXR [76] and CYP3A4 enhancer/promoter reporter gene plasmid pGL4-CYP3A4 (7830Δ7208-364) [77] have been described previously. Metridia luciferase expression plasmid pMetLuc2control was obtained from Takara-Clontech (Mountain View, CA, USA). Site-directed mutagenesis of the full-length PXR expression plasmid with suitable oligonucleotides designed with NEBaseChanger using Q5 Site-Directed Mutagenesis kit (New England Biolabs, Ipswich, MA, USA) was utilized to generate PXR mutants Q285A, Y306A, S247A, H407A, W223A, F281A, W299A, and F429A. The mutations were confirmed by sequencing. Plasmids were purified using PureYield Plasmid Midiprep System (Promega, Madison, WI, USA).

### 4.5 Cell culture

HepG2 cells (HB-8065, lot number 58341723, ATCC, Manassas, VA) were cultivated at 37 °C, 5% CO_2_ in MEM, which was supplemented with 10% FBS, 2 mM glutamine, 100 U/ml penicillin and 100 µg/ml streptomycin. HepG2 cells were originally obtained at passage 74, propagated and used in the experiments between passages 93 and 104. In chemical treatments, regular FBS was replaced by dextran-coated charcoal-treated FBS. Cells were routinely checked for contamination with mycoplasma by PCR (VenorGeM Classic, Minerva Biolabs, Berlin, Germany).

### 4.6 Transient transfections

Transient batch transfection with HepG2 was conducted using 0.6 µl JetPEI transfection reagent per well in a final volume of 25 µl (Polyplus, Illkirch, France). Per well, 0.27 µg pGL4-CYP3A4(-7830Δ7208-364) luciferase reporter gene plasmid, 0.01 µg Metridia luciferase plasmid pMetLuc2-control and 0.03 µg of expression plasmids encoding human PXR or PXR mutants, were diluted in 150 mM NaCl to a final volume of 25 µl. After at least 24 h incubation, cells were treated for 24 h with 0.1% DMSO, 10 µM rifampicin, 1 µM SR12813 or 10 µM test compounds. Metridia luciferase was measured directly from 10 µl of medium with 100 µl Renilla luciferase assay solution [78] using EnSpire 2300 multimode plate reader (PerkinElmer, Rodgau, Germany) for 0.1 s. For firefly luciferase measurement, cells were lysed with passive lysis buffer (Promega, Madison, WI, USA). 10 µl of lysate was combined with 150 µl firefly luciferase assay solution [76] and activity measured with EnSpire 2300 multimode plate reader for 0.1 s. Results were normalized by dividing firefly luciferase activity by Metridia luciferase activity measured in the same well. Expression of mutants was assessed with Western blot (Supplementary Methods Fig. 1).

## Data availability

Raw trajectories of the MD simulations are freely available at https://doi.org/10.5281/zenodo.6048723; https://doi.org/10.5281/zenodo.6355467; https://doi.org/10.5281/zenodo.6615454.

## Declaration of Competing Interest

The authors declare that they have no known competing financial interests or personal relationships that could have appeared to influence the work reported in this paper.

## Appendix A Supplementary data

Supplementary Movie M1

Supplementary Movie M2

Supplementary data 1

Supplementary data: MSM structures
