# Supplementary material for: Discrepancy in interactions and conformational dynamics of pregnane X receptor (PXR) bound to an agonist and a novel competitive antagonist
Source: Comput Struct Biotechnol J. 2022 Jun 13;20:3004–18. doi: 10.1016/j.csbj.2022.06.020 (PMC9218138; doi:10.1016/j.csbj.2022.06.020)
Supplement: Supplementary data 1 [file mmc1.pdf]

SUPPLEMENTARY INFORMATION

Discrepancy in interactions and conformational dynamics of pregnane X receptor (PXR) bound to an agonist and a novel competitive antagonist

Azam Rashidian [a], Enni-Kaisa Mustonen [b], Thales Kronenberger [a,c,e,f], Matthias Schwab [b,d,e], Oliver Burk [b], Stefan A. Laufer [c,e,f], Tatu Pantsar* [c,g]

[a] Department of Internal Medicine VIII, University Hospital Tuebingen, Otfried-Müller-Strasse 14, Tuebingen DE 72076, Germany.

[b] Dr. Margarete Fischer-Bosch-Institute of Clinical Pharmacology, Stuttgart and University of Tuebingen, Tuebingen, Germany

[c] Department of Pharmaceutical and Medicinal Chemistry, Institute of Pharmaceutical Sciences, Eberhard-Karls-Universität, Tuebingen, Auf der Morgenstelle 8, 72076 Tuebingen, Germany.

[d] Departments of Clinical Pharmacology, and Pharmacy and Biochemistry, University of Tuebingen, Tuebingen, Germany

[e] Cluster of Excellence iFIT (EXC 2180) "Image-Guided and Functionally Instructed Tumor Therapies", University of Tuebingen, 72076 Tuebingen, Germany

[f] Tuebingen Center for Academic Drug Discovery & Development (TüCAD2), 72076 Tuebingen, Germany

[g] School of Pharmacy, Faculty of Health Sciences, University of Eastern Finland, Yliopistonranta 1, 70210 Kuopio, Finland.

*Correspondence: mailto:tatu.pantsar@uef.fi

Phone: +358 50 388 1062

Content

Supplementary Methods..4

$CaPO_4$ transfections and protein analysis of mutants.

Supplementary Figure S1...5

Structures of human PXR agonist SJB7 and antagonist SPA70.

Supplementary Figure S2...5

PC1 and PC2 comparison between systems.

Supplementary Table S1..6

The contributions of individual principal components PC1–PC10.

Supplementary Table S2..6

Root-mean-square fluctuations (RMSFs) of individual residues.

Supplementary Figure S3...7

Comparison of B-factors from crystal structures to RMSF-values.

Supplementary Figure S4...7

Additional water-bridged interactions in N-terminus of the α6 region.

Supplementary Figure S5...8

Root-mean-square fluctuations (RMSFs) of ligand heavy atoms.

Supplementary Figure S6...9

Distances of Y306–α3 and F288–α3.

Supplementary Figure S7...9
Hydrophobic interactions of the ligands beyond the hydrophobic subpocket and hydrophobic interaction of F420 with α10/α11.

Supplementary Figure S8...10

Destabilization of αAF-2 in the presence of SRC-1 with compound 100.

Supplementary Figure S9...11

The time-lagged independent component analysis (TICA).

Supplementary Figure S10...11

PXR-LBD conformation in an agonist bound crystal structure (PDB ID: 1NRL).

Supplementary Figure S11...12

Distances associated to PXR conformation of β1-β1’ loop and β4-α6 loop regions.

Supplementary Figure S12……………………………………………..............................12

Hydrophobic interactions of the BAY-1797 beyond the hydrophobic subpocket.

Supplementary Table S3……………………………………………..............................13

Overview of the simulated systems.

Supplementary Figure S13……………………………………………..............................13

Outline of the conducted simulations.

Supplementary Figure S14……………………………………………...........................14

MSM validation.

References………………………………………………...15

Supplementary Methods — *$CaPO_4$ transfections and protein analysis of mutants*

5x10^6 HepG2 cells were seeded a day before transfection in a 10 cm diameter dish. 1 hour prior transfection, culture medium was changed. Aqueous DNA solution of 450 µl was prepared, consisting of 2 µg pMetLuc2control plasmid and 10 µg expression plasmids encoding human PXR or PXR mutants. Total amount of DNA was adjusted to 25 µg with pUC18. 50 µl of $CaCl_2$ was added to each DNA solution, then mixture was added dropwise to an equal volume of 2x HBS while simultaneously mixing before adding dropwise onto cells. Cells were incubated for 5 h before 3 min incubation with 15% glycerol-PBS. Cells were incubated for two days before Metridia luciferase measurement as described in Transient transfections and total protein extraction. Cells were washed with ice-cold PBS and scraped before centrifuging for 5 min at 750 g at 4 °C. Supernatant was discarded and cell pellet resuspendend in protein lysis buffer [1] and incubated on ice for 15 min. Lysate was homogenized with ultrasonication (2x30s) using Bioruptor UCD200 (Diagenode, Liége, Belgium). Protein concentration was determined with bicinchoninic acid method. Protein amount was adjusted to the transfection efficiency as determined by measurement of Metridia luciferase activity. Samples were analyzed on 10% SDS-polyacrylamide protein gels, which was followed by Western blotting to nitrocellulose membrane. Blots were incubated overnight at 4 °C with primary antibodies against human PXR (2 µg/ml; RRID AB_2155076; clone H4417, Perseus Proteomics, Tokyo, Japan) followed by incubation for 1 h at room temperature with peroxidase-conjugated secondary rabbit anti-mouse antibody (0.13 µg/ml; P0260, Dako, Glostrup, Denmark). Detection of chemiluminescence was conducted as described previously [2].

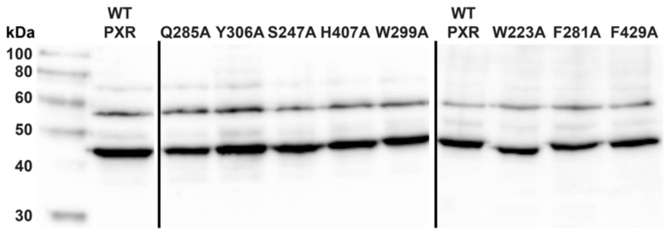

Supplementary Methods Fig. 1. Transfected PXR mutants are expressed in HepG2 cells.

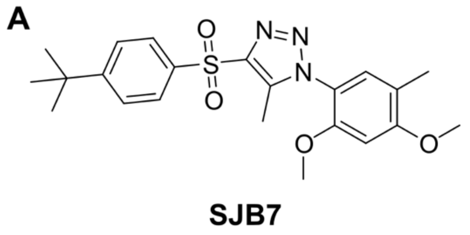

Fig. S1. Structures of human PXR agonist SJB7 (A) and antagonist SPA70 (B).

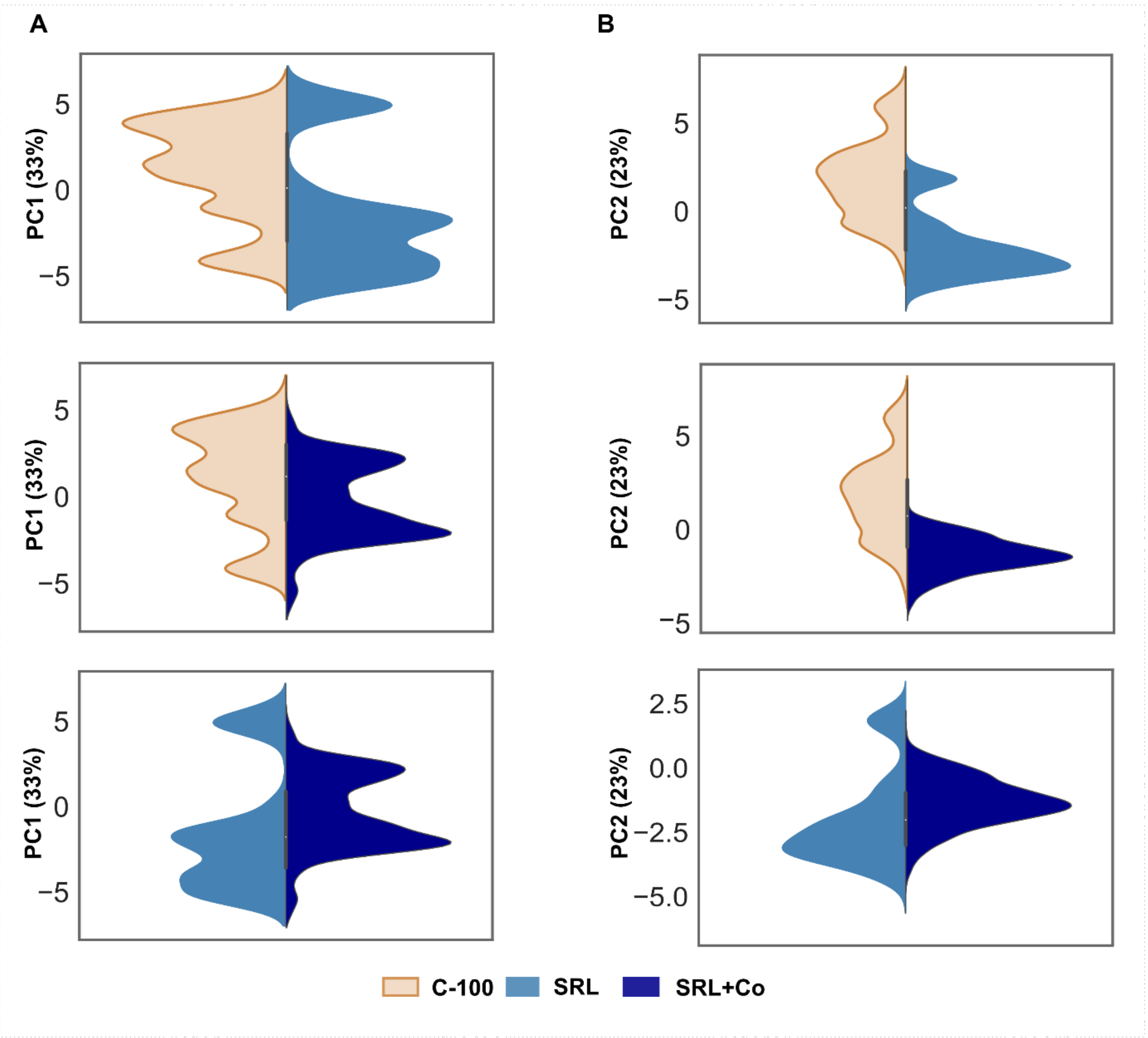

Fig. S2. PC1 and PC2 comparison between systems. In violin plots, a kernel density estimation is used to display the distribution of the data for PC1 (**A**) and PC2 (**B**) in each system. Systems are coloured as following: compound 100 (C-100), light orange; SR12813 (SRL), light blue; SR12813 and SRC1 coactivator peptide (SRL+Co), dark blue.

Table S1. The contributions of individual principal components PC1–PC10.

PC1	PC2	PC3	PC4	PC5	PC6	PC7	PC8	PC9	PC10
33%	23%	9.6%	9.0%	6.0%	5.2%	4.3%	3.6%	3.5%	2.8%

Table S2. Root-mean-square fluctuations (RMSFs) of individual residues (backbone atoms) located on regions associated with the PCA extreme movements. The regions are highlighted with colours according to the colours in Fig. 2C.

RMSF (Å)							
Residue	SRL+Co	SRL	C-100	Residue	SRL+Co	SRL	C-100
α1–α2' loop				β1'–α3 loop			
V177	2.95	2.87	1.79	K226	1.40	1.19	1.04
L178	3.07	3.18	1.78	P227	1.60	1.43	1.16
S179	3.07	3.67	1.98	P228	1.80	1.75	1.46
S180	3.31	3.80	2.21	A229	2.00	2.19	1.67
G181	3.04	3.95	2.40	D230	2.52	2.80	2.08
C182	2.85	4.16	2.16	S231	2.58	2.87	2.02
E183	3.12	3.96	2.09	G232	2.44	3.07	1.93
L184	3.06	3.31	2.09	G233	1.80	2.53	1.43
P185	3.70	3.30	2.31	N234	1.35	1.70	1.04
E186	3.60	3.28	2.61	β4–α6 loop			
S187	3.84	3.90	2.78	D310	1.59	1.57	1.31
L188	3.89	3.82	2.92	T311	2.22	1.93	1.72
Q189	4.02	3.66	3.03	A312	2.94	2.42	2.20
A190	3.52	3.37	2.75	G313	2.99	2.51	2.26
P191	3.33	3.17	2.92	G314	2.66	2.12	1.89
S192	2.72	2.26	2.24	αAF-2			
R193	2.04	1.52	1.91	P423	0.93	1.68	1.69
E194	1.82	1.44	1.89	L424	0.80	1.54	1.58
E195	1.68	1.36	1.80	M425	0.77	1.04	1.34
A196	1.18	0.98	1.42	Q426	0.89	1.17	1.41
β1–β1' loop				E427	0.91	1.28	1.42
G217	1.99	1.96	1.97	L428	0.96	1.04	1.38
E218	2.93	2.70	2.61	F429	1.11	1.28	1.76
D219	3.31	3.03	3.18	G430	1.39	1.81	2.09
G220	2.98	2.93	2.81	I431	1.88	2.02	2.41
S221	2.29	2.41	2.23	T432	2.82	2.94	3.24
				G433	4.53	4.69	4.31
				S434	6.12	6.92	5.75

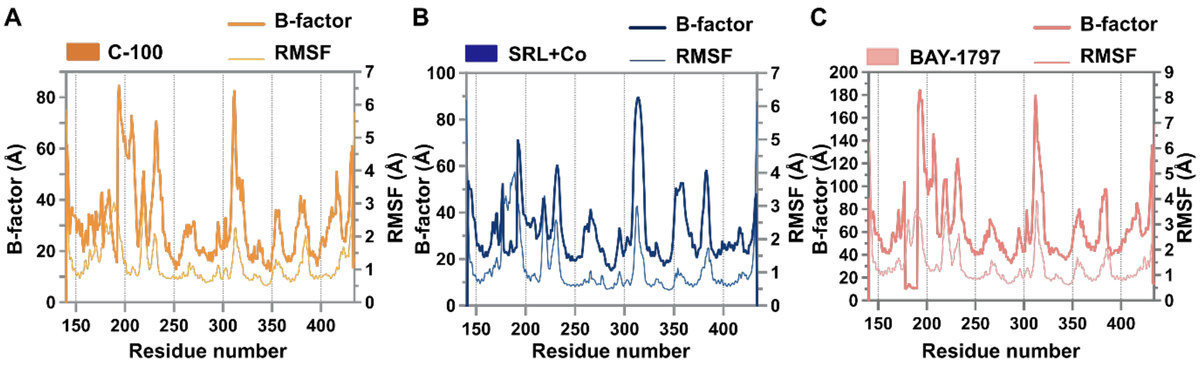

Fig. S3. Comparison of B-factors from crystal structures to RMSF-values. (**A**) B-factor of PXR crystal structure (PDB ID: 4J5W) aligned with the protein RMSF in C-100 system. (**B**) B-factor of PXR crystal structure (PDB ID: 1NRL) aligned with the protein RMSF in SRL+Co system. (**C**) B-factor of PXR crystal structure (PDB ID: 6HTY) aligned with the protein RMSF in BAY-1797 system.

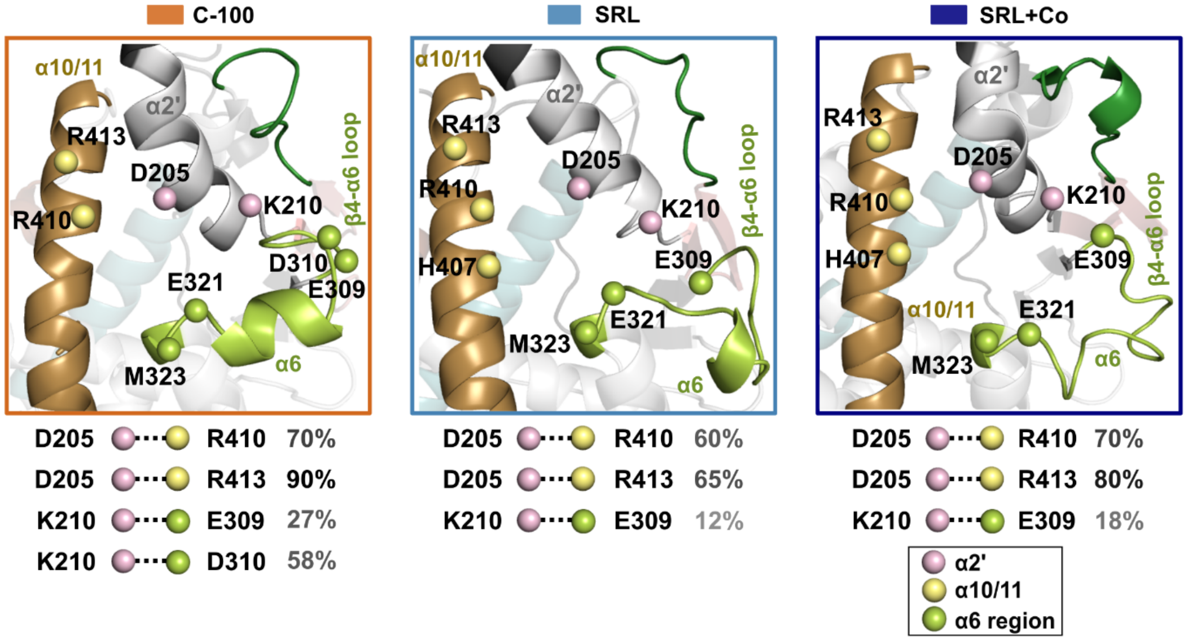

Fig. S4. Additional water-bridged interactions in N-terminus of the α6 region. The additional water bridge interactions in the α6 region (in addition to the observed interactions displayed in Fig. 3B) and their frequencies (cut-off of ≥10%) are shown for each system. The location of the Cα-atom of each interacting residue are indicated with spheres. Spheres belonging to the same helix/region are illustrated with the same colour. The colours of the helices are as in Fig. 1A (main manuscript).

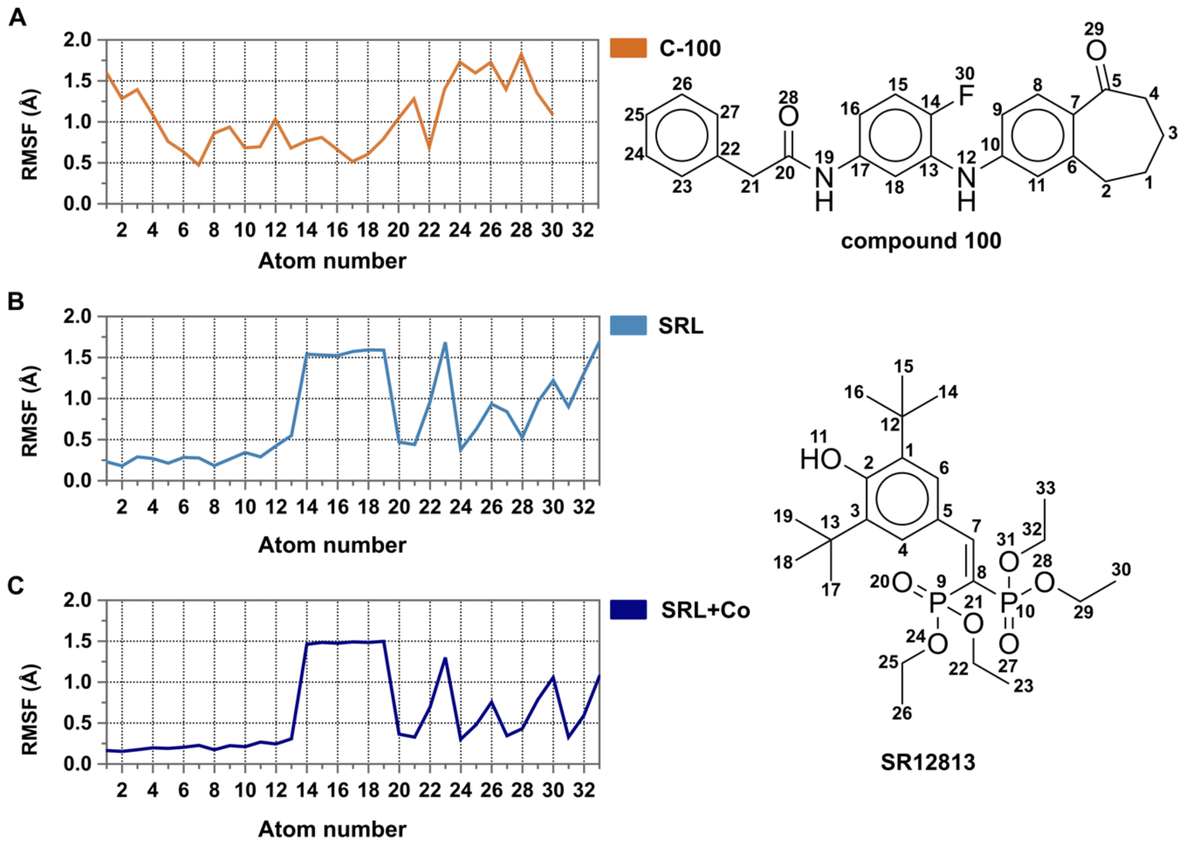

Fig. S5. Root-mean-square fluctuations (RMSFs) of ligand heavy atoms. **(A)** RMSF of compound 100 (C-100), (**B**) SR12813 (SRL) and (**C**) SR12813 with the coactivator (SRL+Co). The atom numbers shown in the plots are illustrated in the 2D structures on the right. Overall, the heavy atoms display low RMSF values (mainly <1Å; flexible substituents <1.7Å), demonstrating the stability of the ligands during the simulations.

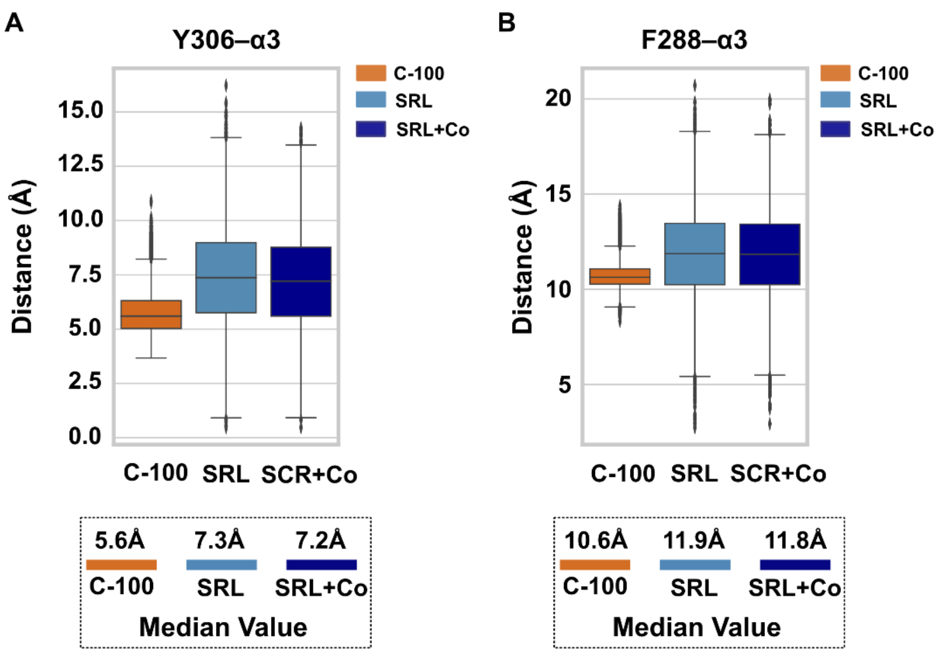

Fig. S6. Distances of Y306–α3 and F288–α3. (A) Distance between the centre of mass of α3 (residues 240–260) and the Cα-atom of Y306 (located in β4). **(B)** Distance between the centre of mass of α3 (residues 240–260) and the Cα-atom of F288 (located in α5).

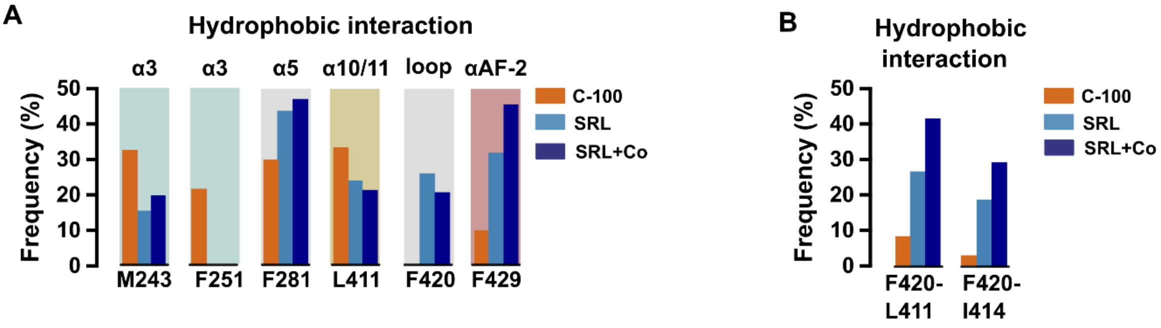

Fig. S7. Hydrophobic interactions of the ligands beyond the hydrophobic subpocket and hydrophobic interaction of F420 with α10/α11. (A) Hydrophobic interactions of the ligands beyond the hydrophobic subpocket. F420 is located on the loop connecting α11 to αAF-2. The boxes are coloured as in PXR-LBD crystal structure in Fig. 1A. **(B)** Hydrophobic interaction of F420 with α10/α11.

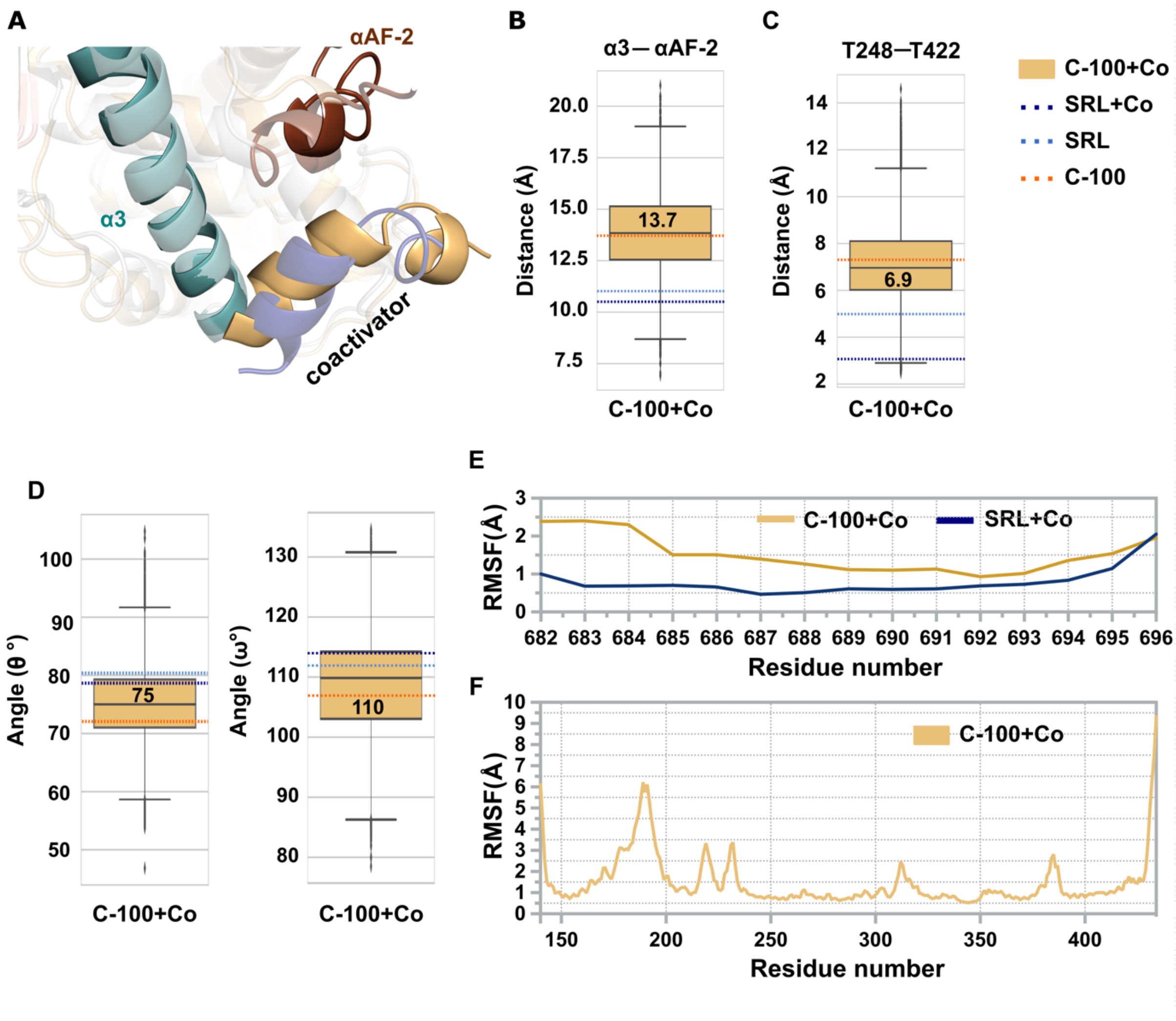

Fig. S8. Destabilization of αAF-2 in the presence of SRC-1 with compound 100. (**A**) Superposition of representative snapshots from C-100+Co over the SRL+Co system. The non-transparent structure is C-100+Co, and transparent colours represent SRL+Co system. (**B**) Movement of αAF-2 in C-100+Co resembles C-100 system. Box plot represents the distribution of distance between centre of mass of α3-helix (residues 240–260) and αAF-2 (residues 423–430). The values in the boxes represent the median of C-100+Co. The dashed lines represent the median value of the of C-100 system (orange dashed line); SRL system (light blue dashed line); SRL+Co system (dark blue dashed line) (see also Fig. 5). (**C**) Distance between sidechain oxygen atoms of T248 and T422 are shown in box plot. (**D**) θ and ω define the angle consisting of N404, F281, F429 and N404, F281, T422, respectively where F281 is the apex. Box plots display the distribution of θ and ω angles. (**E**) Root-mean-square fluctuations (RMSFs) of the SRC-1 backbone atoms display higher values in C-100+Co, especially in its N-terminal part, highlighting its instability with compound 100. (**F**) Root-mean-square fluctuation of the PXR-LBD backbone in C-100+Co.

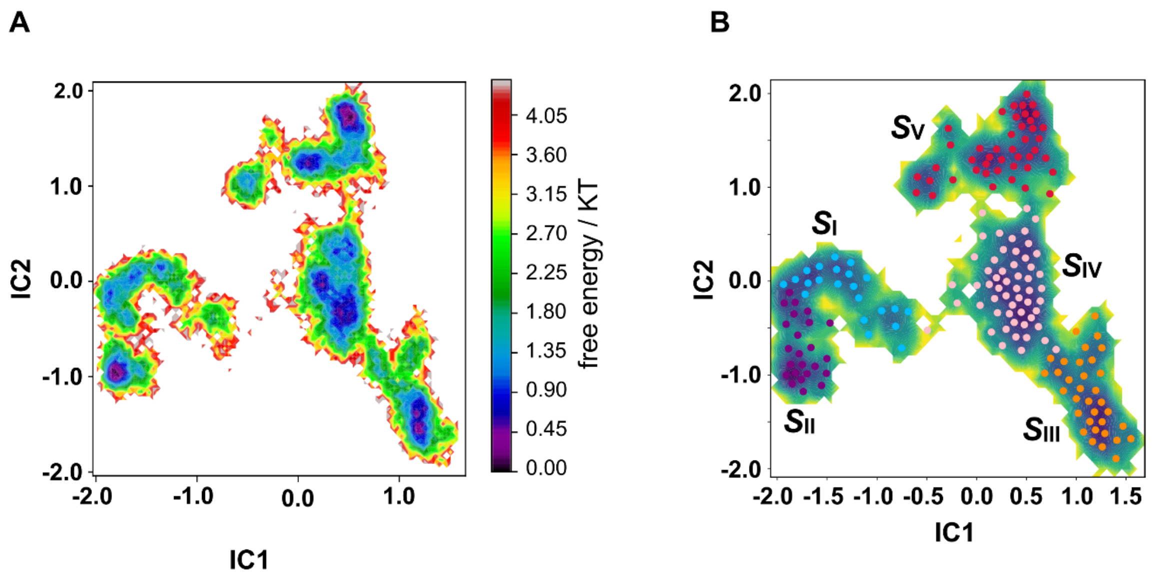

Fig. S9. **The time-lagged independent component analysis (TICA). (A)** Pseudo free energy map of distribution in C-100 projected on TICA components 1 (IC1) and 2 (IC2). **(B)** Separation of the five metastable states by PCCA++. Each individually coloured cluster corresponds to one metastable state ($\boldsymbol{S}_{\mathbf{I-V}}$). The colour code for each cluster is as in Fig. 6.

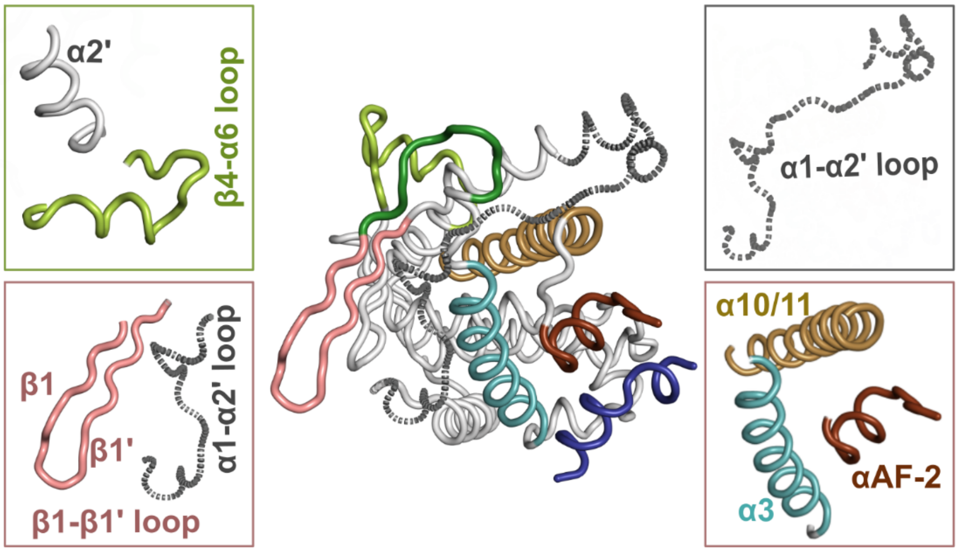

Fig. S10. PXR-LBD conformation in an agonist bound crystal structure (PDB ID: 1NRL). Individual substructures are illustrated as follows: α1-α2' loop, grey dashed line; β-β1' loop, salmon; β4-α6 loop, light green; β1'-α3 loop, dark green; α-AF-2, dark brown.

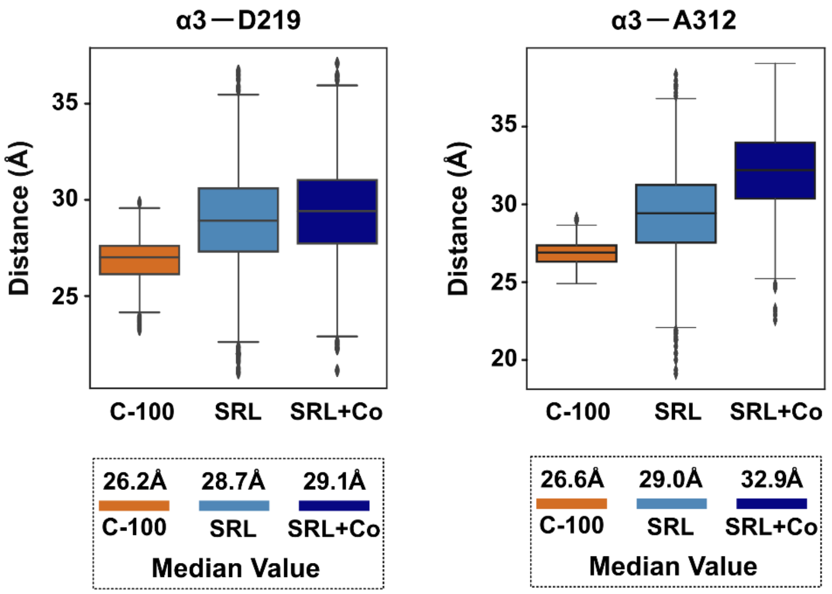

Fig. S11. Distances associated to PXR conformation of β1-β1' loop and β4-α6 loop regions. **(A)** The distance between D219 (apex β1-β1' loop) and α3 (centre of mass of the residues 240–260). This distance is associated the conformational preference in the β1-β1' loop as α3-helix is stable in the simulations: longer distances indicate more extended configuration of this loop, more far away from α3-helix. **(B)** The distance between A312 (located on β4-α6 loop) and α3 (centre of mass of the residues 240–260). This distance is associated the conformational preference in the in β4-α6 loop.

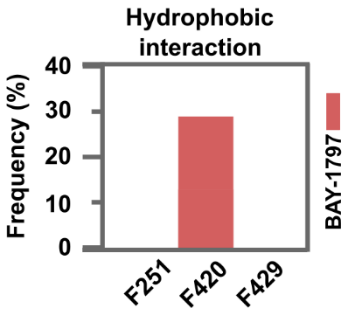

Fig. S12. Hydrophobic interactions of BAY-1797 beyond the hydrophobic subpocket. F420 is located on the loop connecting α11 to αAF-2.

Table S3. Overview of the simulated systems.

PDB ID	Compound	Co-activator	MD simulation time
1NRL	SR12813	SRC-1	10 µs
1NRL	SR12813	-	20 µs
4J5W	100	-	30 µs
4J5W	100	SRC-1	10 µs
6HTY	BAY-1797	-	10 µs

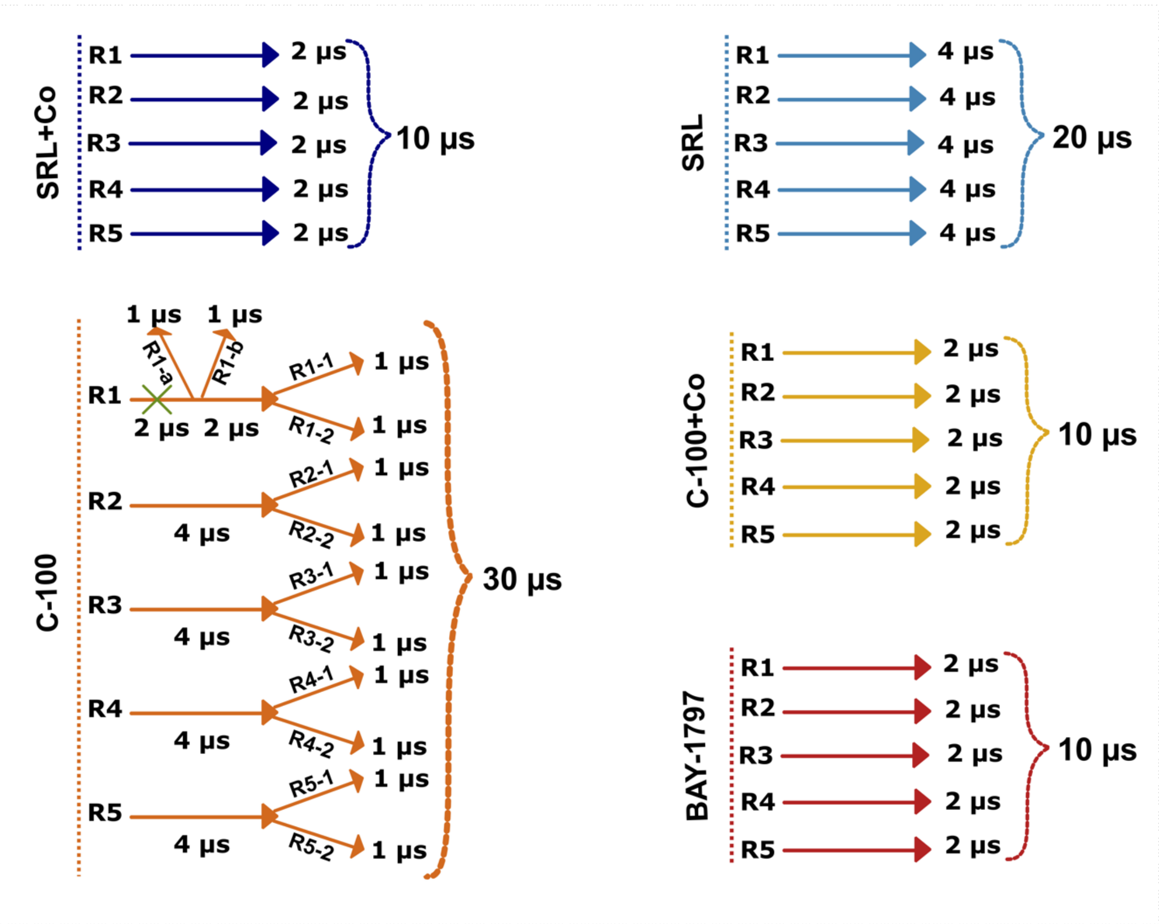

Fig. S13. Outline of the conducted simulations. Fig. S11. Outline of the conducted simulations. This figure illustrates the replicas (R stands for replica) generated and run for each system. In total, seventeen individual replicas were run for C-100 and five replicas for the other systems. Lack of ligand stability in the beginning of the R1 of C-100 led to the generation of more replicas from this trajectory, and the first 2 µs of R1 was excluded from the analysis (denoted here by the green cross sign). Moreover, to obtain additional sampling for C-100,

additional two 1 μs replicas were generated from the output conformations of the original replicas for C-100.

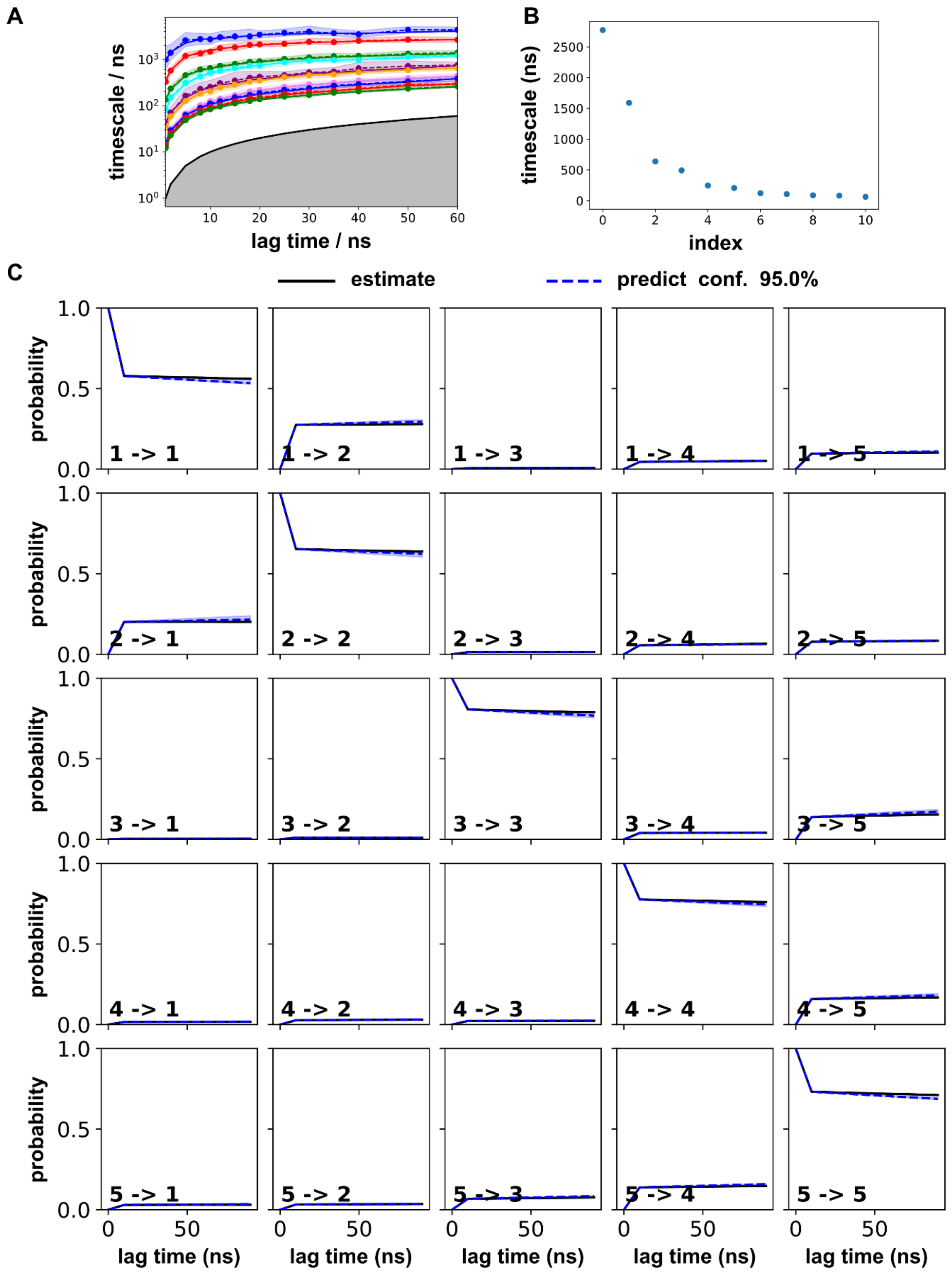

Fig. S14. MSM validation. (**A**) The implied timescales are converged at the used lag time of 10 ns**.** (**B**) The spectral analysis of the timescale separation. A five-state model was selected. (**C**) The Chapman-Kolmogorov test of the MSM shows that MSM (black line) follows the observed trajectory (blue dashed line, with shaded error estimate).

References

[1] Sundqvist A, Bengoechea-Alonso MT, Ye X, Lukiyanchuk V, Jin J, Harper JW, et al. Control of lipid metabolism by phosphorylation-dependent degradation of the SREBP family of transcription factors by SCF(Fbw7). Cell Metab 2005;1:379–91. https://doi.org/10.1016/j.cmet.2005.04.010.

[2] Jeske J, Bitter A, Thasler WE, Weiss TS, Schwab M, Burk O. Ligand-dependent and -independent regulation of human hepatic sphingomyelin phosphodiesterase acid-like 3A expression by pregnane X receptor and crosstalk with liver X receptor. Biochem Pharmacol 2017;136:122–35. https://doi.org/10.1016/j.bcp.2017.04.013.
